# Mouse mammary stem cells express prognostic markers for triple-negative breast cancer

**DOI:** 10.1186/s13058-015-0539-6

**Published:** 2015-03-04

**Authors:** Kelly J Soady, Howard Kendrick, Qiong Gao, Andrew Tutt, Marketa Zvelebil, Liliana D Ordonez, Jelmar Quist, David Wei-Min Tan, Clare M Isacke, Anita Grigoriadis, Matthew J Smalley

**Affiliations:** Division of Breast Cancer Research, Breakthrough Breast Cancer Research Centre, The Institute of Cancer Research, 237 Fulham Road, London, SW3 6JB UK; MRC Molecular Haematology Unit, Weatherall Institute of Molecular Medicine, University of Oxford, John Radcliffe Hospital, Headley Way, Oxford, OX3 9DS UK; European Cancer Stem Cell Research Institute and Cardiff School of Biosciences, Cardiff University, Hadyn Ellis Building, Maindy Road, Cardiff, CF24 4HQ UK; Breakthrough Breast Cancer Research Unit, Guy’s Hospital, Great Maze Pond, London, SE1 9RT UK; Department of Research Oncology, King’s Health Partners AHSC, Life Sciences and Medicine, King’s College London, Guy’s Campus, London, SE1 1UL UK; Institute of Medical Biology, 8A Biomedical Grove, #06-06 Immunos, Singapore, 138648 Singapore

## Abstract

**Introduction:**

Triple-negative breast cancer (TNBC) is a heterogeneous group of tumours in which chemotherapy, the current mainstay of systemic treatment, is often initially beneficial but with a high risk of relapse and metastasis. There is currently no means of predicting which TNBC will relapse. We tested the hypothesis that the biological properties of normal stem cells are re-activated in tumour metastasis and that, therefore, the activation of normal mammary stem cell-associated gene sets in primary TNBC would be highly prognostic for relapse and metastasis.

**Methods:**

Mammary basal stem and myoepithelial cells were isolated by flow cytometry and tested in low-dose transplant assays. Gene expression microarrays were used to establish expression profiles of the stem and myoepithelial populations; these were compared to each other and to our previously established mammary epithelial gene expression profiles. Stem cell genes were classified by Gene Ontology (GO) analysis and the expression of a subset analysed in the stem cell population at single cell resolution. Activation of stem cell genes was interrogated across different breast cancer cohorts and within specific subtypes and tested for clinical prognostic power.

**Results:**

A set of 323 genes was identified that was expressed significantly more highly in the purified basal stem cells compared to all other cells of the mammary epithelium. A total of 109 out of 323 genes had been associated with stem cell features in at least one other study in addition to our own, providing further support for their involvement in the biology of this cell type. GO analysis demonstrated an enrichment of these genes for an association with cell migration, cytoskeletal regulation and tissue morphogenesis, consistent with a role in invasion and metastasis. Single cell resolution analysis showed that individual cells co-expressed both epithelial- and mesenchymal-associated genes/proteins. Most strikingly, we demonstrated that strong activity of this stem cell gene set in TNBCs identified those tumours most likely to rapidly progress to metastasis.

**Conclusions:**

Our findings support the hypothesis that the biological properties of normal stem cells are drivers of metastasis and that these properties can be used to stratify patients with a highly heterogeneous disease such as TNBC.

**Electronic supplementary material:**

The online version of this article (doi:10.1186/s13058-015-0539-6) contains supplementary material, which is available to authorized users.

## Introduction

Breast cancer is a highly heterogeneous disease broadly classified on the basis of clinical parameters such as size, grade and node status, as well as histopathological criteria, primarily expression of estrogen receptor (ER), progesterone receptor (PR), and human epidermal growth factor receptor 2 (HER2) [1]. While defined targeted therapeutic strategies have been developed for patients with ER^+^/PR^+^ and HER2^+^ diseases, chemotherapy is currently the mainstay of systemic treatment for triple-negative (ER^−^/PR^−^/HER2^−^) breast cancer (TNBC) patients, which represents approximately 20% of all breast cancers [2]. Clinically, TNBC encompasses a heterogeneous group of aggressive tumours with poor prognosis [1,3-7], partly due to high recurrence within the first years and limited targeted therapy options. Although chemotherapy is often initially beneficial in these tumours, especially in the neoadjuvant setting, many TNBCs have a high risk of relapse [8]. Since there is currently no means of predicting which TNBC will relapse, identification of subpopulations of TNBC that are most at risk is vital for the clinical management of these breast cancer patients.

Strong evidence is emerging supporting the hypothesis that cancer stem cells with similar features to normal tissue stem cells are resistant to standard chemotherapy and drive tumour regrowth after therapy finishes [9]. We hypothesised that biological properties of normal stem cells are reactivated in tumour cells to facilitate metastasis. Genes expressed in stem cells of the normal mammary gland might therefore carry prognostic information for relapse and metastasis in breast cancer. However, the development of such gene sets depends on the ability to isolate highly pure stem cells for analysis.

The mammary epithelium consists of two main layers, the luminal and basal layers. The luminal layer consists of ER- cells (mainly proliferative progenitors) and ER+ cells (mainly non-proliferative differentiated cells). The basal layer consists of myoepithelial cells (MYOs) and mammary stem cells (MaSCs), the latter characterised by their robust outgrowth activity in the cleared fat pad transplant assay. The relationship between these populations is summarised in Additional file 1A. Previous studies have analysed total basal breast epithelial cells, without further purification of the minority stem cell fraction [10] or used a dye label-retention strategy to identify asymmetrically dividing cells (putative stem cells) in non-adherent mammosphere cultures [11]. Only one previous study has attempted to freshly purify basal stem cells and compare their gene expression profile to MYOs [12]; however, that study identified only four genes expressed >2-fold more highly in stem cells compared to MYOs, and none of these achieved statistical significance. Here, we have defined the first gene signature specific for highly purified, freshly isolated MaSCs and further enriched the stem cell specificity by excluding basal-associated genes common to both the stem and myoepithelial populations. Pathway analysis revealed that this signature was enriched in genes associated with cell migration, adhesion and tissue morphogenesis. Single cell resolution gene expression analysis showed that the stem cell population included cells that expressed both epithelial- and mesenchymal-associated genes. Strikingly, when the expression of the stem cell gene signature was interrogated in two large independent TNBC cohorts, tumours with an activated stem cell signature showed a higher propensity to relapse in the first years after diagnosis in comparison to TNBC with lower activation scores for the stem cell gene signature. In contrast, in three large independent ER+ breast cancer data sets, an activated stem cell signature identified tumours least likely to metastasise. The prognostic power of the stem cell gene signature when applied to expression profiling of total tumour material implies that in poor prognosis TNBC the cancer stem cell-like genetic programme is not restricted to a minority cell population but rather is driving the behaviour of the bulk of tumour cells.

Our findings show that the biology of normal MaSCs, as reflected in their gene expression profiles, is highly relevant for understanding the drivers of aggressive disease in TNBC.

## Methods

### Preparation of mammary epithelial cells for flow cytometry

**Figure 1:**
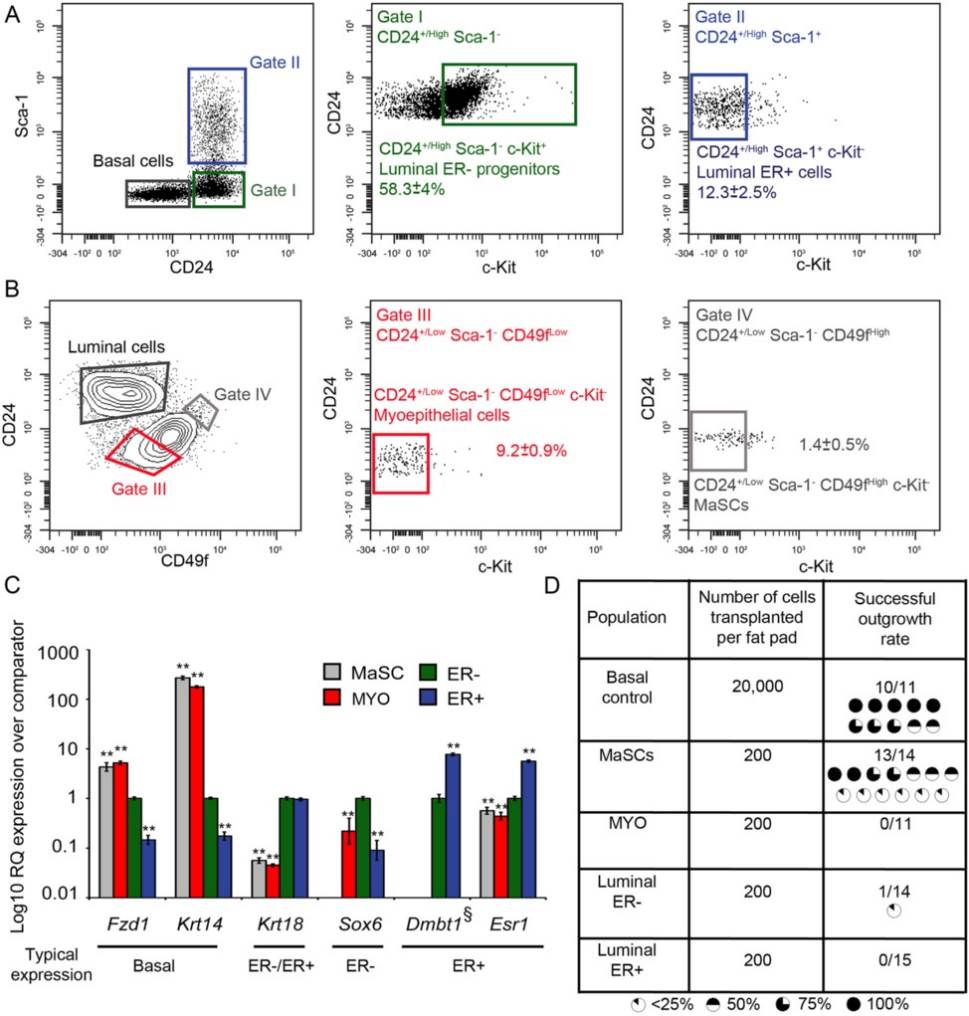
**Isolation of high-purity basal mammary stem cells. (A, B)**. Flow cytometry of mouse mammary epithelial cells isolated from 8- to 10-week-old virgin FVB mice and stained with antibodies against CD24, Sca-1, CD49f and c-Kit. **(A)** Isolation of the luminal ER- progenitor (Gate I) and luminal ER+ cell populations (Gate II) on the basis of CD24 and Sca-1 expression followed by c-Kit as previously described [13]. **(B)** Isolation of basal myoepithelial (MYO; Gate III) and mammary stem cells (MaSCs; Gate IV), from the same sort data as in **(A)**, on the basis of CD24 and CD49f expression followed by c-Kit as previously described [13]. Only epithelial cell data is shown. For the full gating hierarchy see Additional file 1. **(C)** qrtPCR gene expression analysis of mammary epithelial lineage-specific genes. Data expressed as mean fold expression (±95% confidence intervals) over comparator population (luminal ER- cells) in three independent isolates of each cell population. Statistical significance was determined according to [74]. ^**^ *P* <0.01 relative to comparator. ‘Typical expression’ indicates the populations expected to have the highest levels of expression of the genes based on previous findings [13]. ^§^ *Dmbt1* expression was undetectable in all isolates of MaSC and MYO populations. **(D)** Results of low-dose transplantation of mammary subpopulations into cleared fat pads. The number of successful outgrowths, the total number of fat pads transplanted and the extent each outgrowth filled the fat pads, as indicated by the pie chart symbols, are shown. ER, estrogen receptor; qrtPCR, quantitative real-time rtPCR.

All animal work was carried out under UK Home Office project and personal licences following local ethical approval by the Institute of Cancer Research Animal Ethics Committee and in accordance with local and national guidelines. Single cells were prepared from fourth mammary fat pads of 8- to 10-week-old virgin female FVB mice as described [13] and stained with anti-CD24-FITC, anti-Sca-1-APC, anti-CD45-PE-Cy7, anti-CD49f-PE-Cy5 and anti-c-Kit-PE. Mammary epithelial cell subpopulations were defined as shown in Figure 1 and Additional file 1.

### Cleared mammary fat pad transplantations

Transplantation of primary mouse mammary epithelial cells was carried out as described [13]. Sorted cells were transplanted at 200 cells per fat pad into the cleared fat pads of 21-day-old syngeneic FVB mice over three independent sort and transplant sessions for each population. Positive control transplants of total basal cells at 20,000 cells per fat pad were also included in each session. Fat pads were analysed by whole-mounting eight weeks after transplantation.

### RNA isolations and gene expression analysis by quantitative real-time rtPCR (qrtPCR)

Freshly sorted primary cells were lysed in RLT buffer (Qiagen, Crawley, West Sussex, UK) and stored at −80°C. Total RNA was extracted using an RNeasy MiniElute or MicroElute Kit (Qiagen), according to the manufacturers’ instructions. qrtPCR reactions were performed as previously described use Taqman probes (see Additional file 2) [14]. Results either were calculated using the ΔΔCt method and expressed as the mean fold gene expression difference in three independently isolated cell preparations over a comparator sample with 95% confidence intervals, or, for single cell experiments, presented as raw 1/Ct values.

### Immunofluorescent staining of cells sorted on to slides

Samples of 50 to 200 cells were sorted directly on to poly-L-lysine-coated slides, air dried, and stored at −20°C. The cells were fixed in 1:1 methanol acetone for 5 minutes at −20°C (keratin 14 (K14)/keratin 18 (K18)) or 4% paraformaldehyde (ERα and keratin 15 (K15)/vimentin (Vim)) for 30 minutes at room temperature (RT). Slides were rinsed twice in phosphate-buffered saline (PBS) and permeabilised by incubation with 0.5% PBS/TritonX-100 for 10 minutes. Slides were washed twice in PBS then once in immunofluorescence buffer (IFF; 1% w/v bovine serum albumen (Invitrogen, Life Technologies, Paisley UK), 2% v/v foetal calf serum (FCS, Invitrogen) in PBS) before incubation with primary antibodies (mouse anti-K15 clone LHK15, 1:200 dilution, #ab80522 Abcam, Cambridge, UK; rabbit anti-vimentin EPR3776, 1:500 dilution, #2707-1 Epitomics/Abcam; mouse anti-K14 clone LLOO2, 0.26 ug/ml, #ab7800 Abcam; mouse anti-K18 clone Ks18.04, 1:5 dilution, #61028 Progen Biotechnik, Heidelberg, Germany; mouse anti-ERα clone ID5, 9.9 ug/ml, #M7047 Dako, Cambridge, UK) for 60 minutes at RT. Cells were washed with PBS three times for 5 minutes each before the Alexa-conjugated secondary antibodies (Alexa-488 and/or Alexa-555, each at 1:500, Invitrogen) were applied for 60 minutes at RT. The slides were washed three times for 5 minutes each in 0.01% PBS/DAPI, rinsed in water and mounted and coverslipped with Vectashield (Vector Laboratories, Peterborough, UK). Images were captured using a Leica TCS-SP2 microscope with images collected in three channels using Leica LCS software (Leica Microsystems, Wetzlar, Germany). ‘No First Antibody’ controls were used to set PMT levels. Controls using only one first antibody with both second antibodies were used to confirm lack of cross-reactivity of second antibody staining.

### Immunocytochemical staining of mammary tissue

Paraffin-embedded tissue sections on poly-L-lysine-coated slides were dewaxed in xylene (2 × 5 minutes) and rehydrated by washing in decreasing concentrations of ethanol: 2 × 3 minutes in 100% ethanol, 1 × 3 minutes 95% ethanol and 1 × 3 minutes 75% ethanol. Antigen retrieval was carried out by incubating the slides in preheated citrate buffer (99.9°C, pH6; Thermo Fisher Scientific, Loughborough, UK) for 20 minutes. Slides were then left to cool for 30 minutes at RT. Slides were then incubated with a peroxidase-blocking solution (Vim and smooth muscle actin (SMA): 3% hydrogen peroxide, CK14: 1 in 60 hydrogen peroxide v/v in methanol) for 10 minutes at RT, followed by three 5-minute washes in washing buffer (Vim and CK14: 0.1% Tween in Tris-buffered saline (TBS), SMA: PBS). The slides were blocked with serum diluted in wash buffer (Vim and SMA: normal goat serum, CK14: MOM diluents, MOM kit, Vector Laboratories) for 45 minutes at RT. The serum block was removed and slides were incubated immediately with the primary antibody in serum/wash buffer overnight at 4°C. Vim was detected using a goat polyclonal antibody (Santa Cruz SC-7557, Santa Cruz Biotechnology, Santa Cruz, CA, USA) at 1:300 dilution, SMA using a rabbit polyclonal (Abcam, #ab5694), and CK14 using a mouse monoclonal antibody (Abcam, #ab7800). Unbound primary antibody was removed by three 5-minute washes in wash buffer and then the slides were then incubated for 1 hour at RT with the secondary antibody in serum/wash buffer (Vim and SMA: anti-rabbit biotinylated (Dako), CK14: anti-mouse (MOM kit)). The positive signal was amplified by incubating the slides for 30 minutes at room temperature with the Avidin-Biotin Complex (ABC) kit (Vector Laboratories), made up 30 minutes before it was applied, then positivity was visualised by incubating the slides with the DAB+ Chromogen reagent (EnVision™ kit, Dako). The reagent was applied to the slides for 5 minutes, and then removed by three washes with wash buffer. The slides were counterstained in haematoxylin for 60 seconds, followed by a wash in running water for 5 minutes. The slides were dehydrated by washing in increasing concentrations of alcohol, placed in xylene for 2 × 5 minutes and then mounted with a glass coverslip.

### Affymetrix transcriptome analysis

RNA was isolated from three independent myoepithelial and seven independent MaSC isolations. Samples were submitted to the UCL Genomics facility (UCL Institute of Child Health, London, UK) for amplification and hybridisation to the Mouse Genome 430 2.0 Affymetrix array (Affymetrix, High Wycombe, UK). Total RNA was amplified using the NuGEN Ovation Pico WTA System (Nugen, Leek, The Netherlands). Resulting double-stranded cDNA was fragmented and labelled using the Affymetrix Genechip WT Terminal Labelling kit. Affymetrix Mouse Genome 430 2.0 chip arrays were hybridised and scanned according to manufacturer’s instructions.

Expression data were normalised and summarised by robust multi-array analysis (RMA) using the Affymetrix package in R. Probesets mapping to unknown or multiple genes were removed from analysis. Probesets were used for two class unpaired comparison using significance analysis of microarray (SAM) R package [15], genes that were enriched or depleted in the MaSC population compared to the myoepithelial population were determined by a local false discovery rate (FDR) <5%. For comparison to data from Kendrick *et al.* [16], all CEL files were RMA normalised together and two class unpaired SAM using a local FDR of 5% was applied to each population compared to the MaSC population. Probesets were also used for a multiclass SAM [16] to determine if their mean expression was different across the four mammary epithelial cell subpopulations. Hierarchical cluster based on Pearson’s correlation with average linkage was performed in the software package Cladist of the ROCK database [17,18]. Pathway analysis was performed using the DAVID KEGG pathway analysis tool and the ROCK pathway analysis tool [17-20]. All analyses were carried out using default settings. Pathway gene sets with an enrichment score of FDR of 5% were considered significantly enriched. Overlaps between gene sets were visualised using VENNY [21]. MIAME-compliant data have been uploaded to ArrayExpress with the accession number E-MTAB-2741.

### Single cell resolution gene expression analysis

Single MaSCs were subjected to PCR essentially as described previously [22]. See Additional file 2 for details of primer sequences. To generate cDNA, a single MaSC was sorted into thin-walled 0.2 ml 96-well plates (Corning Life Sciences, Amsterdam, The Netherlands) containing first-strand buffer (Superscript III buffer (Invitrogen), 0.5% Nonidet P40 (Pierce, Thermo Fisher Scientific, Cramlington, UK), 1 mM dNTP mixture (Invitrogen), 1 mM DTT (Invitrogen), SuperRNaseIN (Ambion, Life Technologies, Paisley, UK)), 3.4 nM MO_4_d(T) primer and *A. thaliana* spike mRNAs LTP4, LTP6 and TIM (Stratagene, Agilent, Stockport, Cheshire, UK) added at serial tenfold pg/μl dilutions. Single cells sorted into first-strand buffer were snap-frozen in liquid nitrogen and lysed at 65°C. Primer was allowed to anneal at 45°C before addition of Superscript III reverse transcriptase and incubation at 45°C. The reaction was inactivated at 65°C for 10 minutes. Unannealed primer was digested by exonuclease I (New England Biolabs, Hitchin, UK) with 6.7 mM MgCl2 at 37°C. Removal of the RNA template and polyadenylation of the cDNA were carried out concurrently by the addition of RNaseH (New England Biolabs), 1.5 mM dATP (Invitrogen) and 30 units terminal deoxynucleotidyl transferase (TdT, Promega, Southampton, UK) at 37°C. Four microlitres of polyadenylated cDNA was used as template for PCR amplification in 1× ExTaq buffer (TaKaRa Clontech, St Germain-en-Laye, France), 0.65 mM dNTP (Invitrogen), 8.25 μM MO_4_d(T) primer, 5 units ExTaq (TaKaRa) by incubating at 94°C for 1 minute, 50°C for 2 minutes, and 72°C for 2 minutes to allow second-strand synthesis. Subsequently, 35 cycles of amplification were performed by incubating at 94°C for 30 seconds, 60°C for 30 seconds, and 72°C for 2 minutes. The first round of amplification was performed in triplicate, after which the amplified cDNA was pooled. A second round of amplification was performed in duplicate using 2 μl of the pooled amplified cDNA as template in 1× ExTaq buffer (TaKaRa), 0.2 mM dNTP (Invitrogen), 2 μM T7-MO_4_ primer and 5 units ExTaq (TaKaRa). Thirty-five cycles of amplification were performed by incubating at 94°C for 30 seconds, 60°C for 30 seconds, and 72°C for 2 minutes. The amplified cDNA was again pooled before use.

In preliminary tests, monitoring of the amplification of the spiked control RNA (*LTP4* added at 10^−2^ pg, *LTP6* at 10^−3^ pg and *TIM* at 10^−4^ pg; these values correspond to 8,400, 900 and 90 molecules of RNA respectively) in single cell samples from the CommaDβgeo [23] mammary epithelial cell line demonstrated that using the procedure amplification was linear and preserved relative abundance of transcripts, although a small amount of variation was inherent to the second round of amplification (Additional file 3A). Furthermore, when qrtPCR for seven genes (*Gapdh, Ubc, Jag1, Jag2, Wnt4, Wnt5a and Wnt10a;* selected on the basis of probe availability) was carried out on amplification material from 16 single CommaD cells, 16 groups of 10 CommaD cells and on unamplified cDNA collected from the bulk population, the mean of the expression levels of the single cells was not significantly different from the mean of expression in the 16 groups of 10 cells or to expression levels in the unamplified bulk cDNA. This analysis confirmed that relative expression levels were preserved upon amplification from a single cell with a strong correlation in relative expression levels obtained when comparing single cell and 10-cell amplified material (R = 0.98) and single cell amplified and whole population unamplified material (R = 0.95) (Additional file 3B; R values calculated in Excel).

### Breast cancer data set collection

Three TNBC cohorts were used in this study. A total of 579 cases described by Karn and colleagues (Karn579) was downloaded from the Gene Expression Omnibus, accession ID GSE31519 [24]. The second TNBC dataset, referred to as Guy’s107, comprised the TNBC extracted from the recent GSE40267 study [25]. The third TNBC dataset (Lehmann) is a compilation of TNBC extracted from 24 gene expression profiling data sets (including 228 cases of the Karn579 cohort) all performed on the Affymetrix hg-u133a platform (ETABM158, MDA133, GSE1456, GSE5327, GSE5847, GSE7390, GSE1561, GSE11121, GSE2034, GSE2603, GSE20194, GSE2990, GSE3494, GSE2109, GSE12276, GSE18864, GSE7904, GSE16446, GSE19615, GSE31519, GSE10780, GSE13787, GSE6596 and GSE5460) [26] on which we carried out RMA pre-processing followed by a combat normalisation to reduce batch effect [27]. For ER+ tumours, we retrieved the NKI295 [28], TRANSBIG [29] and the GSE2990 [30] data sets and extracted those cases which were termed positive for ER by immunohistochemistry, resulting in 226, 134, 149 samples for NKI295, TRANSBIG and GSE2990, respectively. Clinico-pathological features for each of these data sets have been previously published in the original manuscripts referenced above, except for the Lehmann set, which is provided here as Additional file 4. Details of ethical approval for patient material can be found within the original publications relating to each data set.

### Analysis of MaSC gene signature in breast cancer transcriptional profiles

The mouse MaSc gene signature derived from the SAM was converted to a human gene list using Biomart ID conversion (Ensembl Genes 72// mus musculus genes GRCm38.p1). To establish the overall activity of the MaSc genes signature in human breast cancers, we applied our previously published Denoising Algorithm based on Relevance network Topology (DART) [31], which identifies genes within a signature with highly correlated expression levels and uses these to infer molecular pathway activity. We also tested median centring the gene expression of the data set and establishing the activation of the whole MaSC gene signature by averaging the relative expression values for all genes for each tumour. The ‘DART’ activation score or averaged gene expression for each sample in each cohort were determined and log-rank tests were performed dichotomising the samples using either top tertile or median cutoffs, depending on the data set. Kaplan-Meier plots were generated for each data set to provide a visualisation of survival stratification.

### Breast cancer subtype classification

Centroid classification for the PAM50 molecular breast cancer subtypes were performed as described previously [25]. PAM50 and IntClust classifications were retrieved from the original publications [5,32]. TNBC subtypes describe by Lehmann and colleagues were established using the online TNBC-type program [33]. To determine the four TNBC subtypes described by Burstein and colleagues [34], centroids for each subtype were extracted and correlation analysis performed.

### Statistics

All statistical tests were two-sided unless otherwise noted. Hypergeometric testing was used to establish the significance of overlap between two gene lists. All analyses were performed within the statistical R environment [35] unless otherwise noted.

## Results

### Isolation of high-purity basal mammary stem cells

To isolate MaSCs with high purity, a flow cytometry approach using the markers CD24, CD49f, c-Kit, Sca-1 and CD45 was used [13] (Figure 1 and Additional file 1B,C). Total epithelial cells were identified by expression of CD24 and Sca-1 as previously described [13] (Figure 1A). Luminal ER- progenitors (Gate I) and luminal ER+ cells (Gate II) were further identified on the basis of previously defined staining patterns (CD24^+/High^Sca-1^−^c-Kit^+^ and CD24^+/High^Sca-1^+^c-Kit^−^, respectively) [13]. The basal epithelial population was partitioned into MYOs (Gate III; CD24^+/Low^Sca-1^−^CD49^Low^c-Kit^−^) and MaSC (Gate IV; CD24^+/Low^Sca-1^−^CD49^High^c-Kit^−^) populations (Figure 1B) [13]. As previously [36], a high threshold for a cell being ‘CD49f^High^’ was set (5% most strongly stained CD49f-positive cells in the basal population) in order to achieve a very high purity of MaSCs (which amounted to 1.4 ± 0.5% of the total mammary epithelium; mean ± standard deviation (SD), n = 3 sorts).

To confirm that the gating strategy isolated MaSCs, MYOs, luminal ER- cells and luminal ER+ cells, the populations were sorted and characterised by qrtPCR gene expression analysis, staining of cytospins and *in vivo* functional (transplant) assays. qrtPCR analysis of expression of a panel of previously defined cell type-specific genes [16] demonstrated that the populations isolated by the sorting strategy had the expected pattern of gene expression. CD24^+/Low^Sca-1^−^CD49^High^c-Kit^−^ and CD24^+/Low^Sca-1^−^CD49^Low^c-Kit^−^ cells expressed high levels of *Fzd1* and *Keratin14* (*Krt14*; the gene encoding K14) (basal genes). Both CD24^+/High^Sca-1^−^c-Kit^+^ and CD24^+/High^Sca-1^+^c-Kit^−^ cells expressed the pan-luminal gene *Keratin18* (*Krt18*; the gene encoding K18), while CD24^+/High^Sca-1^−^c-Kit^+^ cells expressed the luminal ER- gene *Sox6* and CD24^+/High^Sca-1^+^c-Kit^−^ cells expressed the luminal ER+ genes *Dmbt1* and *Estrogen receptor 1* (*Esr1*; the gene encoding ERα*)* (Figure 1C).

Staining of cells from the populations sorted on to slides for K14, K18 or ERα showed that the populations which were positive for these antigens were also those which had shown the highest levels of *Krt14*, *Krt18* or *Esr1* gene expression respectively by qrtPCR analysis (Table 1). One hundred percent of CD24^+/Low^Sca-1^−^CD49^High^c-Kit^−^ MaSCs and 84% of MYOs cells were K14 positive. The vast majority of these cells were also K18 negative, although two rare double-positive MaSCs were observed (Additional file 5). In contrast, all luminal cells were K18 positive and K14 negative, with strongest K18 staining seen in the CD24^+/High^Sca-1^+^c-Kit^−^ luminal ER+ population. Immunofluorescence staining for ERα confirmed that the CD24^+/High^Sca-1^+^c-Kit^−^ cells were 100% ERα positive. There was a small number (4%) of ERα-positive cells in the CD24^+/High^Sca-1^−^c-Kit^+^ luminal ‘ER-’ population, also in agreement with previous data [14].

**Table 1:** **Results of staining of cells sorted on slides for keratins 14 (K14) and 18 (K18) and ERα**

**Population**	**Antigen (number of cells counted)**	**Staining (%)**		
		**-**	**−/+**	**+**
MaSCs	K14 (106)	0	19	81
	K18 (106)	98	2	0
	ERα (62)	100		0
MYOs	K14 (79)	16	48	35
	K18 (79)	100	0	0
	ERα (60)	100		0
Luminal ER-	K14 (128)	100	0	0
	K18 (128)	2	52	46
	ERα (65)	96		4
Luminal ER+	K14 (118)	100	0	0
	K18 (118	0	20	80
	ERα (60)	0		100

Cells were either double-stained for K14/K18 or single-stained for ERα and then counterstained with DAPI. Examples of negative (−), weak (−/+) and positive (+) staining and of double-stained MaSCs are shown in Additional file 5. ERα staining was only scored as negative or positive. ERα, estrogen receptor alpha; MaSC, mammary stem cell; MYO, myoepithelial cell.

To finally validate the sorting strategy using the cleared mammary fat pad transplant assay, the MaSC, MYO, luminal ER+ and luminal ER- populations were sorted and transplanted into cleared fat pads at 200 cells per fat pad over three independent cell sorts and transplantations. After 8 weeks, glands were harvested, whole-mounted, carmine stained to enable visualisation of outgrowths and scored. The results (Figure 1D) showed that only the CD24^+/Low^Sca-1^−^CD49^High^c-Kit^−^ MaSC population had the ability to repopulate a mammary fat pad with high efficiency and, when taken in conjunction with the qrtPCR and cell-staining data, confirmed that the sorting strategy was able to isolate MaSCs, MYO, luminal ER- and luminal ER+ cells.

### MaSCs have a distinct gene expression profile to myoepithelial and luminal cells

MaSCs are localised in the basal cell layer of the mammary epithelium. While MaSCs exclusively show repopulation capacity, they share a number of features with the other, most numerous, cell type of the basal cell layer, the MYOs. For instance, both MaSCs and MYOs express K14, although *Krt14* gene expression is slightly higher in MaSCs than MYOs [37]. Direct comparison between luminal cell gene expression and MaSC gene expression, even when using highly purified populations, will identify genes associated with the basal cell layer as a whole, as well as the MaSC genes. Therefore, the comparison between the highly purified MaSC and myoepithelial populations is essential in identifying genes solely characterising the MaSC population.

**Figure 2:**
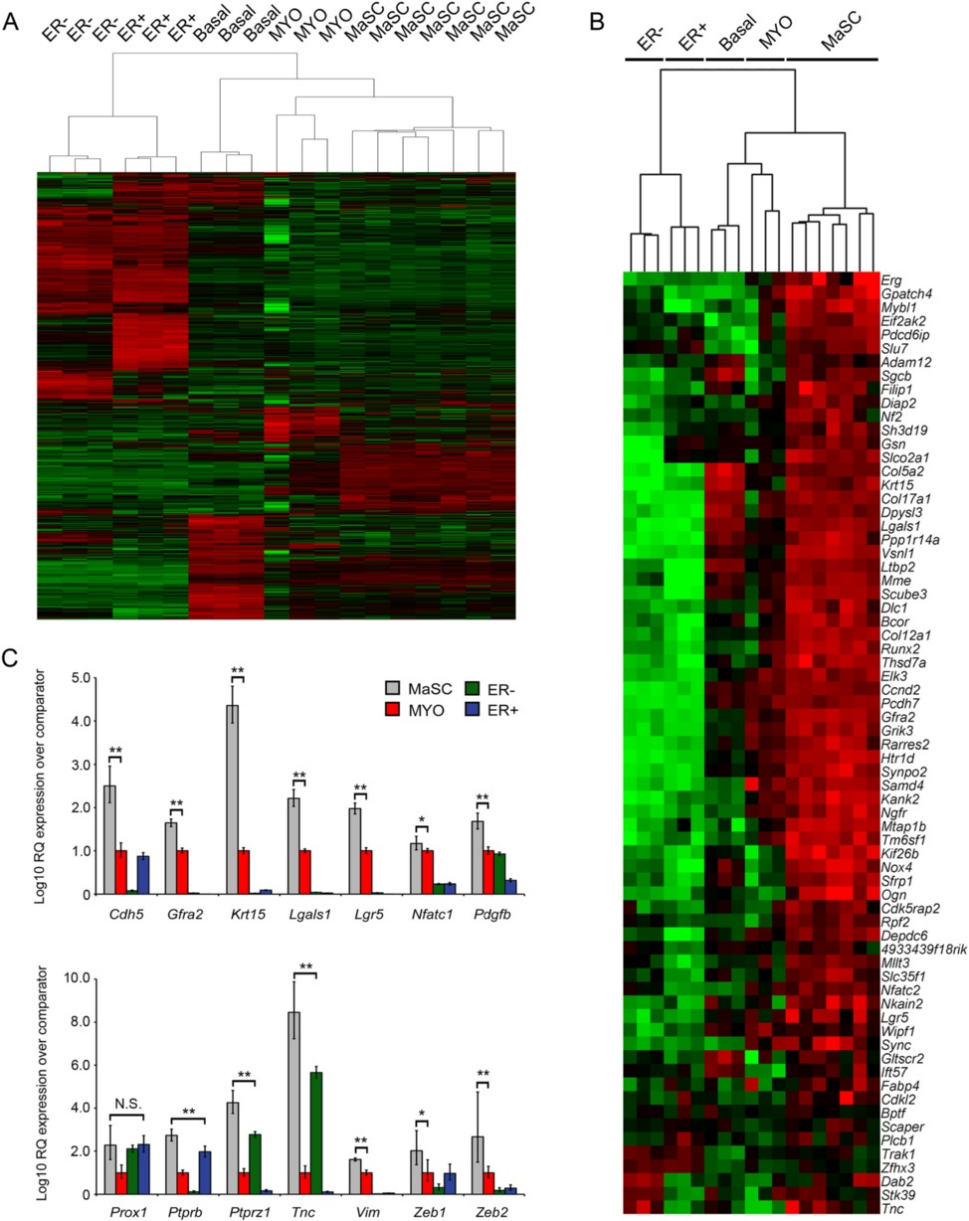
**MaSCs have a distinct gene expression profile. (A)** Unsupervised hierarchical clustering heatmap of genes differentially expressed between MaSC, MYO, total basal, luminal ER- (ER-) and luminal ER+ (ER+) mammary epithelial subpopulations. Expression values represent median-centred value for each probeset. **(B)** Unsupervised hierarchical clustering of microarray expression data of the 69 top MaSC signature genes (Table 2) across the MaSC, MYO, total basal, ER- and ER+ populations. **(C)** qrtPCR validation of MaSC genes of interest identified by whole transcriptome microarray analysis. Expression of *Cdh5, Gfra2, Krt15, Lgals1, Lgr5, Nfatc1, Pdgfb Prox1, Ptprb, Ptprz1, Tnc, Vim, Zeb1, Zeb2* in luminal ER-, luminal ER+, MYO and MaSC unamplified RNA determined by qrtPCR analysis. Data expressed as mean relative fold expression (±95% confidence intervals) over comparator population (luminal ER- cells) in three independent isolates of each cell population. Statistical significance was determined according to [74]. ^**^ *P* <0.01; ^*^ *P* <0.05; N.S not significant, relative to the next highest expressing population indicated by brackets. ER, estrogen receptor; MaSC, mammary stem cell; MYO, myoepithelial cell; qrtPCR, quantitative real-time rtPCR.

We had previously profiled purified luminal ER+ and luminal ER- cells and the total basal epithelial population, which is dominated by MYOs [16]. To extract MaSC-specific but not common ‘basal’ genes or a MYO-dominated gene set, gene expression using Affymetrix microarray of highly purified CD24^+/Low^Sca-1^−^CD49^High^c-Kit^−^ MaSCs and CD24^+/Low^Sca-1^−^CD49^Low^c-Kit^−^ MYOs was carried out. These data were integrated with our previous work on the total basal and two separated luminal ER- and luminal ER+ cells populations [16] analysed on the same Affymetrix gene expression platform. Analysis of the distribution of the raw data from both the previous arrays and our new analyses showed no batch effects between the data sets that might have skewed results (Additional file 6A). Unsupervised hierarchical clustering of gene expression data (Figure 2A) demonstrated that the individual samples of the total basal cells (CD24^-/Low^Sca-1^−^) from our previous analysis [16] and the new MYO (CD24^+/Low^Sca-1^−^CD49^Low^c-Kit^−^) and MaSC (CD24^+/Low^Sca-1^−^CD49^High^c-Kit^−^) samples were more similar to each other than to the two luminal populations. Notably, however, individual samples from the total basal, MYO and MaSC populations clustered with each other. In particular, the seven MaSC samples formed a distinct branch within the basal cluster. This suggested that the transcriptome of the MaSC samples was distinct to those of both the luminal, total basal and MYO populations. By applying a series of two-class unpaired SAM comparisons [15], genes significantly upregulated in the MaSCs relative to all other populations were determined as follows. First, MaSC genes significantly upregulated in the MaSCs compared to the MYOs were identified, using a FDR of <5% and a fold change cutoff of 1.5. Such an approach will inevitably identify some genes that are expressed in MaSCs at a higher level than in myoepithelial cells but are in fact, when all cell populations are considered, much more highly expressed in luminal populations. This is partly due to the relative, rather than absolute, quantitative nature of the approach but also likely to result from ‘lineage priming’ [38]. Therefore, the MaSC gene expression data were also separately compared to the luminal ER- (CD24^High^Sca-1^−^) and ER+ (CD24^High^Sca-1^+^) populations. Applying an FDR <5% and a cutoff of 1.5 fold change, 323 genes were identified as significantly upregulated in MaSCs relative to all the other populations and considered to be MaSC-specific (Additional file 7). Of these 323 genes, 69 were expressed >2.5 fold higher in the MaSCs relative to both the MYO and combined luminal populations (Table 2; Figure 2B).

**Table 2:** **Expression of top 69 MaSC signature genes expressed >2.5 fold higher and <5% FDR relative to expression in both myoepithelial and luminal cells, ordered by gene symbol**

	**Relative to myoepithelial expression**		**Relative to luminal expression**	
**Gene symbol**	**Fold change**	**FDR (%)**	**Fold change**	**FDR (%)**
*4933439F18Rik*	2.60	2.44	4.93	0
*Adam12*	5.40	2.45	2.77	0
*Bcor*	2.77	3.37	2.87	0
*Bptf*	3.98	3.49	7.57	0
*Ccnd2*	2.83	3.49	8.89	0
*Cdk5rap2*	2.64	0.58	3.33	0
*Cdkl2*	2.66	1.75	2.86	0
*Col12a1*	3.76	2.85	4.18	0
*Col17a1*	6.72	3.49	2.51	0
*Col5a2*	2.97	0.58	3.55	0
*Dab2*	3.53	3.49	2.51	0
*Depdc6*	2.63	2.25	3.88	0
*Diap2*	2.54	2.85	2.97	0
*Dlc1*	2.54	3.37	5.57	0
*Dpysl3*	2.68	3.49	4.92	0
*Eif2ak2*	2.75	2.85	2.83	0
*Elk3*	2.68	3.49	3.26	0
*Erg*	2.64	2.85	2.55	0
*Fabp4*	3.59	2.44	2.50	0
*Filip1*	2.55	4.30	2.68	0
*Gfra2*	3.21	0.37	10.55	0
*Gltscr2*	2.51	3.49	2.71	0
*Gpatch4*	2.63	3.90	3.28	0
*Grik3*	3.29	0.96	4.53	0
*Gsn*	2.73	2.44	2.98	0
*Htr1d*	2.78	2.02	5.85	0
*Ift57*	2.90	0.00	3.18	0
*Kank2*	3.19	1.59	4.23	0
*Kif26b*	4.02	0.00	3.64	0
*Krt15*	3.38	1.75	3.15	0
*Lgals1*	3.01	0.00	6.39	0
*Lgr5*	7.76	0.00	2.94	0
*Ltbp2*	2.87	0.00	6.94	0
*Mllt3*	2.88	2.06	5.10	0
*Mme*	3.17	1.59	4.29	0
*Mtap1b*	6.74	2.08	3.64	0
*Mybl1*	3.05	2.06	3.17	0
*Nf2*	3.14	3.37	3.46	0
*Nfatc2*	2.81	1.54	6.35	0
*Ngfr*	2.95	3.49	3.16	0
*Nkain2*	3.14	2.23	3.78	0
*Nox4*	5.04	0.58	2.69	0
*Ogn*	5.79	1.71	2.68	0
*Pcdh7*	2.68	3.49	5.51	0
*Pdcd6ip*	2.93	2.85	3.77	0
*Plcb1*	2.87	1.10	2.72	0
*Ppp1r14a*	2.61	3.49	3.14	0
*Rarres2*	2.90	2.06	2.87	0
*Rpf2*	3.95	4.30	3.36	3.98
*Runx2*	2.60	2.44	2.95	0
*Samd4*	4.94	1.75	5.49	0
*Scaper*	3.69	3.49	2.68	1.86
*Scube3*	3.71	0.58	4.39	0
*Sfrp1*	3.51	1.55	5.20	0
*Sgcb*	2.61	1.99	2.79	0
*Sh3d19*	2.94	2.44	5.46	0
*Slc35f1*	5.18	2.06	2.61	0
*Slco2a1*	3.38	2.08	4.18	0
*Slu7*	2.80	3.56	2.54	0
*Stk39*	3.20	3.49	4.15	0
*Sync*	2.58	2.44	3.86	0
*Synpo2*	3.56	1.59	4.10	0
*Thsd7a*	3.41	3.90	2.50	0
*Tm6sf1*	3.83	2.44	2.65	0
*Tnc*	21.41	0.00	2.65	0
*Trak1*	2.96	4.30	4.86	0
*Vsnl1*	5.30	0.00	5.35	0
*Wipf1*	2.75	0.58	3.98	0
*Zfhx3*	3.32	2.06	3.82	0

MaSC, mammary stem cell; FDR, false discovery rate.

Fourteen genes (*Cdh5*, *Gfra2*, *Krt15*, *Lgals1*, *Lgr5*, *Nfatc1*, *Pdgfb*, *Prox1*, *Ptprb*, *Ptprz1*, *Tnc*, *Vim*, *Zeb1*, *Zeb2*) were selected to validate the Affymetrix-obtained MaSC gene expression by qrtPCR analysis of independent RNA samples isolated in three mammary cell preparations and sorts. These genes were selected on the basis of their identification by other authors as of potential interest in embryonic development and/or adult normal and cancer stem cell biology [10,11,39-46]. Importantly, the RNA used for this validation was unamplified. Validation was carried out on the luminal ER- (CD24^+/High^Sca-1^−^c-Kit^+^), luminal ER+ (CD24^+/High^Sca-1^+^c-Kit^−^), MYO (CD24^+/Low^Sca-1^−^CD49^Low^c-Kit^−^) and MaSC (CD24^+/Low^Sca-1^−^CD49^High^c-Kit^−^) subpopulations. In agreement with the microarray data, 13 of 14 genes were significantly more highly expressed in the MaSCs, compared to all other populations (Figure 2C). The only exception was *Prox1*, which was more highly expressed in MaSCs than MYOs but was expressed at similar levels in MaSCs and both luminal populations (Figure 2C).

**Figure 3:**
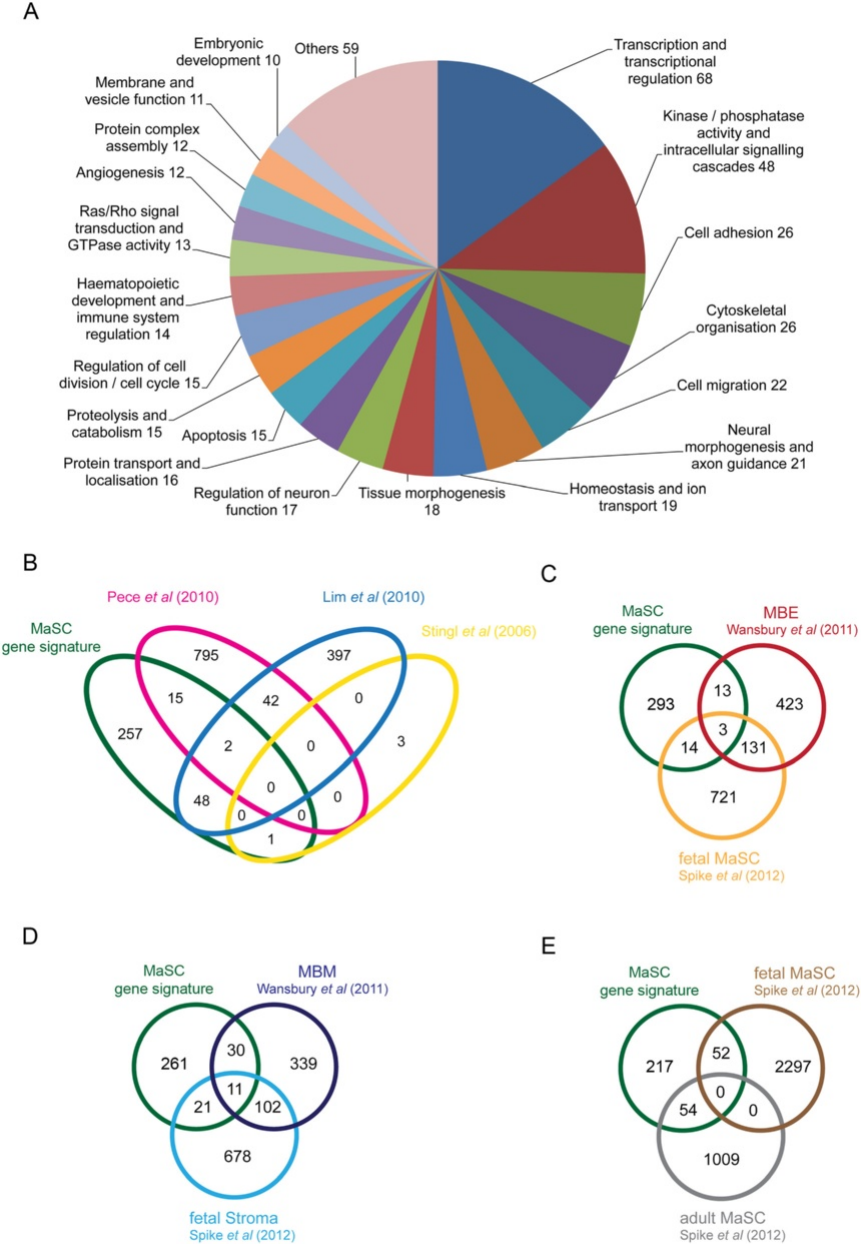
**Analysis of the MaSC gene signature. (A)** Breakdown of GO Bioprocess classifications of MaSC gene signature based on DAVID annotation clustering (Additional file 5). **(B-E)** VENNY analysis of overlap between MaSC signature and published data sets [10-12,39,40] (Additional file 6). **(B)** Overlap with published Stingl, Lim and Pece gene lists of adult stem cell genes. **(C)** Overlap with Wansbury fetal mammary bud epithelium and Spike fetal mammary stem cell genes. **(D)** Overlap with Wansbury fetal mammary mesenschyme and Spike fetal mammary stroma genes. **(E)** Overlap with Spike fetal and adult stem cell gene lists. GO, Gene Ontology; MaSC, mammary stem cell.

To interrogate the 323 MaSC genes for functional associations, analysis of the total MaSC gene list was carried out using the DAVID Gene Ontology (GO) tool and the ROCK database pathway analysis tool [17,19,20] (Figure 3A and Additional file 8). The MaSC gene signature was most highly enriched for genes associated with transcription, intracellular signalling, cell adhesion and cytoskeletal organisation/cell migration/axonal guidance (Figure 3A). Pathway analysis highlighted a number of pathways associated with genes in the signature including, notably, smooth muscle contraction, interactions with the extracellular matrix/integrins and Wnt signalling (Additional file 8).

The top 100 genes most strongly downregulated in the MaSCs compared to the MYOs are shown in Additional file 9, with GO analysis of these genes in Additional file 10. Since many of these genes are not well-annotated, no significant enrichment for a particular pathway was identified. GO analysis also offered only limited insights, although there was enrichment for genes associated with transcription, immune system regulation, apoptosis and haematopoietic development.

A notable absence from the list of MaSC-specific genes was *Procr*, recently suggested as a marker of multipotent MaSCs with epithelial-mesenchymal transition (EMT)-like features [47]. To directly test *Procr* expression in our mammary cell subpopulations, qrtPCR gene expression analysis was carried out on MaSCs, MYOs, luminal ER- and luminal ER+ subpopulations as defined in Figure 1. There was no significant difference in *Procr* expression between MaSCs and MYOs and, indeed, *Procr* expression could be detected in luminal ER+ cells (although at lower levels than in MaSCs; Additional file 6B).

### Comparison of the MaSC signature to previously identified stem cell signatures

Previous studies have identified human and mouse ‘MaSC signatures’ using either freshly isolated cells [10,12] or mammosphere cultures [11], with the caveats noted above. To establish whether any genes were identified common to these studies, gene expression signatures from these studies were overlaid with the signature reported here (Figure 3B-E and Additional file 11). Only one gene was common between our MaSC signature and the genes identified by Stingl and colleagues [12], namely fatty acid-binding protein 4, adipocyte (*Fabp4*). This gene has recently been shown to mark a population of adipocyte progenitors but has not yet been linked functionally to MaSCs [48]. Fifty genes were found in common between our MaSC gene signature and the signature identified by Lim and colleagues [10]. Of the genes expressed in stem cells isolated from mammosphere cultures using a label-retaining strategy [11], 17 were shared with the MaSC signature reported here (Figure 3B).

To assess whether adult MaSCs share gene expression profiles with their more primitive embryonic counterparts, the MaSC signature was compared to two embryonic mammary bud studies. One study profiled the gene expression signatures of the constituent tissues of purified mammary primordia, the mammary bud epithelium (MBE) and mammary bud mesenchyme (MBM) [40]. The other compared CD24^High^CD49f^High^ fetal MaSC (fMaSC) against either CD24^Med/Low/Neg^ fetal stromal cells or adult CD24^Low^CD49f^High^ MaSCs [39].

Only three genes overlapped between our MaSC signature and both embryonic epithelial profiles, namely *Nkain2*, *Mtap7 and Mbp* (Figure 3C; Additional file 11, highlighted in red). However, 27 genes from the MaSC signature were expressed in one of the two profiles. Furthermore, there were 11 adult MaSC genes that overlapped with both previously identified embryonic mesenchymal/stromal profiles, namely *Dab2*, *Ebf3*, *Flt1*, *Klf12*, *Ldb2*, *Ogn*, *Samd4*, *Tek*, *Tfpi*, *Wscd2* and the Riken ORF *9030425E11Rik* (Figure 3D; Additional file 11, highlighted in blue). There were 54 genes in common between our MaSC gene signature and the adult MaSC profile identified by Spike and colleagues (Figure 3E; Additional file 11). Taken together, 109 out of 323 genes (34%) in the MaSC signature have already been associated with adult mammary stem cell features in at least one other study. GO Bioprocess analysis annotates 28 of these 109 genes (*Aebp1, Akap2, Cd36, Cdh5, Cdh13, Cldn5, Cntn2, Col12a1, Col18a1, Col 6a2, Dclk1, Dst, Emcn, Flt1, Gsn, Lgals1, Mtap1b, Myh11, Nexn, Ngfr, Nrp2, Pcdh7, Pecam1, Postn, S1pr1, Sgcb, Thy1, Tns1*) as involved in adhesion, migration and/or cytoskeletal reorganisation, supporting a role in invasion/metastasis. Fifteen of the 109 genes (*Cdh13, Col17a1, Cpne8, Dst, Epas1, Fabp4, Jam2, Krt15, Lmod1, Mical3, Myh11, Ngfr, Ntn4, Ppp1r14a, Tns1*) have been identified in two or more studies in addition to our own, providing further support for their involvement in the biology of this cell type.

### Single cell resolution analysis of MaSCs demonstrates co-expression of epithelial and mesenchymal features

GO analysis had demonstrated that the MaSC gene signature included classes of genes associated with cell migration and invasion. However, population level gene expression analysis may mask important transcriptional or functional heterogeneity within populations being assayed. Therefore, to test the heterogeneity of MaSCs and whether they had the potential to be further divided into functional subtypes, we analysed expression of a subset of MaSC-signature genes in individual MaSCs using a single cell resolution gene expression protocol [22]. The protocol is based on two rounds of PCR amplification of reverse-transcribed RNA. Importantly, it includes spiking input RNA with exogenous RNA of known amounts (from *Arabidopsis thaliana*), both to act as internal controls for ΔΔCt calculations and to monitor linearity of amplification during the procedure. See Methods for details of validation steps used for the protocol.

**Figure 4:**
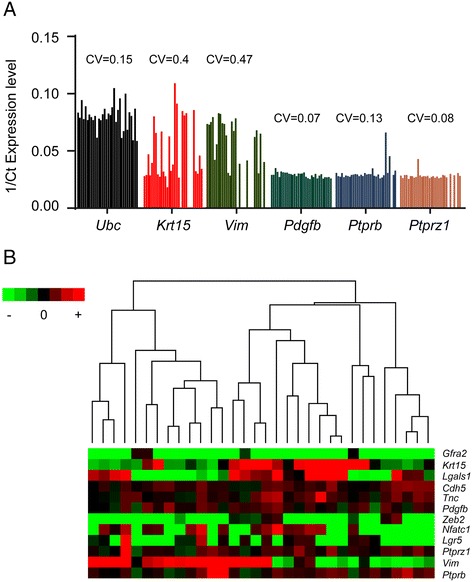
**Individual MaSCs have diverse gene expression patterns. (A)** Coefficients of variation of gene expression between individual cells are variable, with some being highly diverse (*Krt15*, *Vim*) and others more uniform (*Pdgfb, Ptprb, Ptprz1*). Data presented as 1/Ct values. **(B)** Unsupervised hierarchical clustering of median-centred qrtPCR gene expression data on 12 genes from 32 individual MaSCs. Note there are no obvious blocks of similarity of gene expression across the whole panel between the cells. MaSC, mammary stem cell; qrtPCR, quantitative real-time rtPCR.

**Figure 5:**
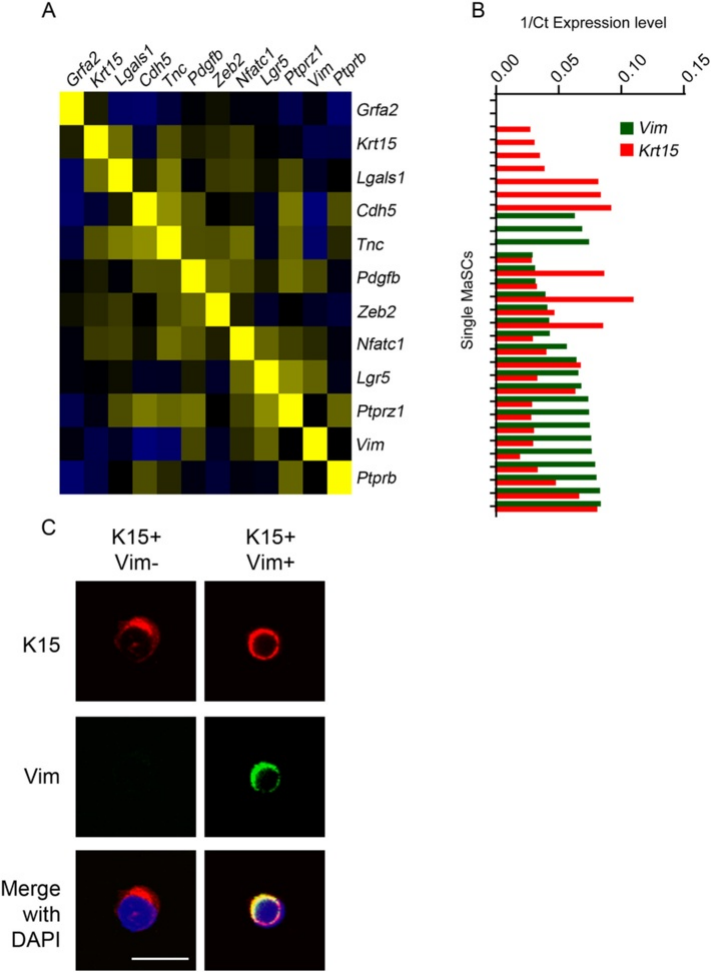
**The MaSC population does not contain distinct subpopulations. (A)** Pearson correlation between gene expression profiles of individual cells showing a low level of correlation in gene expression between cells (see Additional file 12 for details). **(B)** Comparison of *Vim* and *Krt15* gene expression in individual MaSCs by qrtPCR. Data presented as 1/Ct values. **(C)** Examples of K15 single-stained and K15/Vim double-stained MaSCs. Bar = 10 um. K15, keratin 15; MaSC, mammary stem cell; qrtPCR, quantitative real-time rtPCR; Vim, vimentin.

We analysed 32 individual MaSCs by qrtPCR for expression of a panel of 12 genes identified by the Affymetrix microarray analysis as significantly more highly expressed in MaSCs than in the other mammary epithelial populations (Figure 4). Expression of some genes was highly variable across the individual single cells (for example *Krt15* and *Vim*) while for others, expression was more consistent (for example *Pdgfb*, *Ptprb* and *Ptprz1*) (Figure 4A). Although *Krt15* and *Vim* expression defined distinct sets of cells (double positives, single positives and double negatives) (Figure 4B), each pattern of expression of the 12 genes tested was typically unique to each cell, although some patterns were found in two of the cells tested (numbering the cell profiles in Figure 4B from left to right, cells 23 and 24 appear almost identical, as are 30 and 31). Classes of cells with overall gene expression patterns that were similar within classes but very different between the classes were not observed and were not defined by unsupervised hierarchical clustering (Figure 4B). This was further supported by the lack of significance in Pearson correlations between gene (Figure 5 and Additional file 12) and suggests that MaSCs cannot be divided into cell subsets with uniform patterns of gene expression. Rather, individual cells sampled at any particular point in time (co-)express genes associated with a particular phenotype - epithelial-associated or mesenchymal-associated genes for example - in unique patterns and without necessarily expressing the full transcriptional programme of that phenotype.

Epithelial and mesenchymal features of stem cells are of particular interest because EMT has been reported as a characteristic of cancer stem cells and mesenchymal-like features are associated with increased migration and invasive potential [9,49,50]. However, it should be noted that co-expression of epithelial-associated and mesenchymal-associated genes/proteins in the mammary epithelium is not unique to the mammary stem cells. Indeed, the basal cell population in the mammary epithelium, which is >90% MYOs [13], is known to express both mesenchymal and epithelial markers, whether shown by immunofluorescence for example K14, α-SMA and Vim [14,51,52] or gene expression profiling of bulk populations for example *Krt5, Krt14, Mylk, Snai2, Vim* [16]. We confirmed this here by immunohistochemical staining of serial sections of normal mouse mammary gland for K14, SMA and Vim expression. The basal cell layer of the mammary epithelium shows strong expression of K14 and SMA in all cells, and weaker, punctate Vim staining in a subset of cells (Additional file 13A). It is clear that both MaSCs and MYOs co-express epithelial and mesenchymal markers; nevertheless, patterns of co-expression of epithelial- and mesenchymal-associated genes in stem cells in particular are of interest in informing our understanding of the biology of EMT-MET conversions in cancer stem cells. Therefore, the co-expression of *Krt15* (K15), an epithelial-associated gene, and *Vim* (Vim), a mesenchymal-associated gene, and their respective proteins were examined in the single MaSCs in more detail.

1/Ct qPCR expression levels of *Krt15* and *Vim* for each individual cell showed that the majority of cells co-expressed both genes; of the 32 cells analysed, 2 (6.2%) were double negative, 7 (21.9%) expressed *Krt15* but not *Vim*, 3 (9.4%) expressed *Vim* but not *Krt15* and 20 (62.5%) were double positive (Figure 5B). Therefore, more than half of MaSCs do express both genes. Furthermore, when MaSCs were flow sorted on to slides and double stained with antibodies against K15 and Vim (n = 234 cells stained and counted from three separate cell preparations), most cells (92.5 ± 2.5%) were positive for both K15 and Vim. A few cells were positive for only one marker (K15 1.3 ± 1.0%, Vim 4.0 ± 1.0%) or double negative for both proteins (2.2 ± 1.7%). Thus the population variation in expression of *Krt15*/K15 and *Vim*/Vim seen at mRNA level is less evident at the protein level (Figure 5C). Most MaSCs therefore simultaneously express at least some aspects of the epithelial and mesenchymal biological programmes but do not necessarily show either a full epithelial or mesenchymal phenotype.

### The MaSC signature is strongly predictive of distant metastasis-free survival in breast cancer

**Figure 6:**
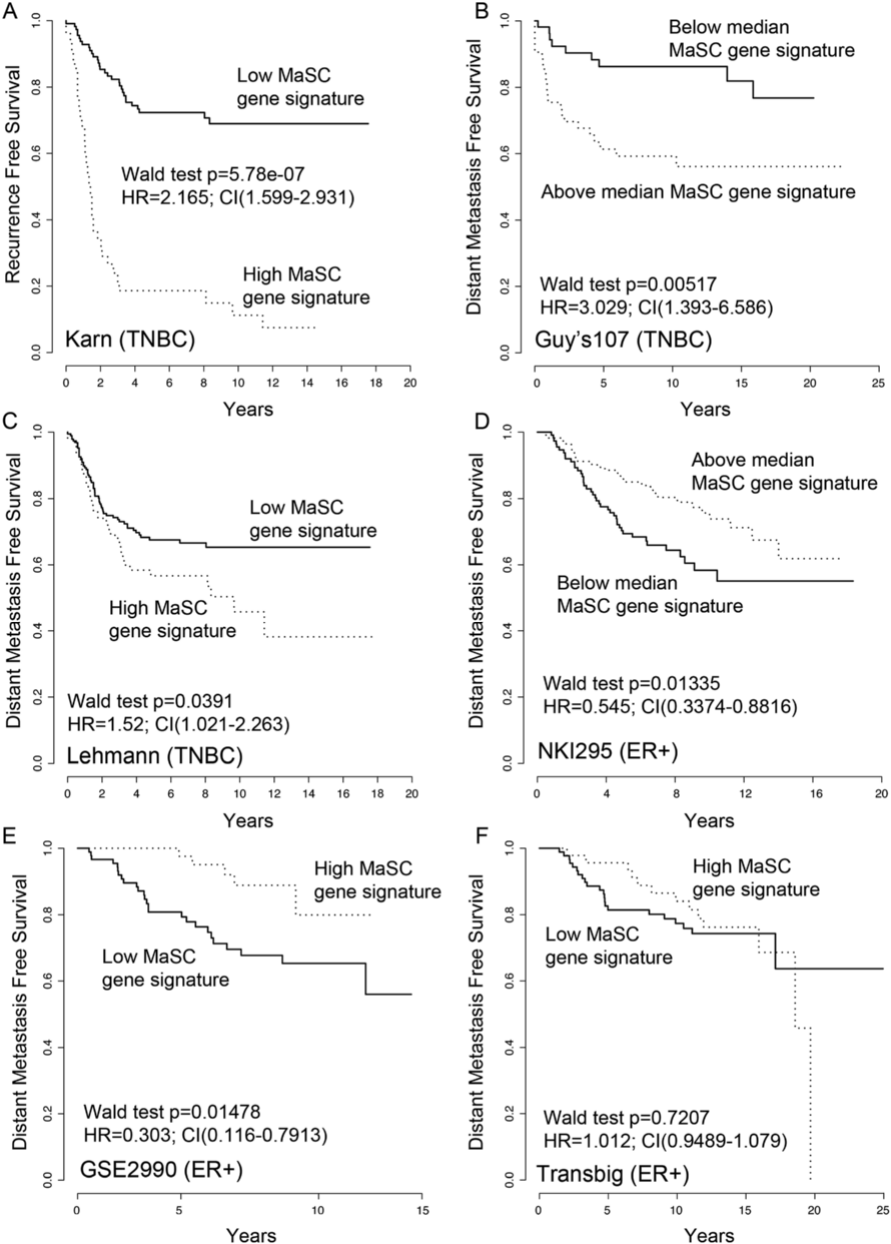
**The MaSC gene signature predicts a significantly shorter DMFS in TNBC.** Kaplan-Meier survival curves showing distant metastasis-free survival (DMFS) in three cohorts of TNBC and three of ER+ breast cancer. Stratification is based on top tertile of samples with high DART activation score for enrichment of MaSC gene signature compared to the rest, with the exception of the Guy’s107 TNBC dataset, which was stratified using the median activation score as a cutoff. **(A)** Karn579 TNBC [24]. **(B)** Guy’s107 TNBC [25]. **(C)** Lehmannn TNBC [26]. **(D)** NKI295 ER+ [28]. **(E)** GSE2990 ER+ [30]. **(F)** TRANSBIG ER+ [29]. DART, Denoising Algorithm based on Relevance network Topology; ER, estrogen receptor; MaSC, mammary stem cells; TNBC, triple-negative breast cancer.

Having established a robust MaSC gene set, we asked whether these genes were expressed in human primary breast cancers and if their expression provided any evidence that the biology of MaSCs as reflected in their gene signature has relevance for breast cancer progression. We initially examined TNBCs as these tumours strongly overlap with the basal-like intrinsic molecular subtype of breast carcinomas, which have previously been associated with a high proportion of stem cell-like cells, both in terms of flow cytometric phenotype and functional assays [9,53,54]. Mouse genes of the MaSC signature were converted to Ensembl human gene identifiers and the activation of the MaSC gene signature in two independent TNBC cohorts was determined. One previously published dataset, the Karn579 dataset, is a compilation of 579 TNBC extracted from several breast cancer cohorts [24]. The second comprises the Guy’s107 TNBC, which includes the TNBC samples from the Guy’s collection [25]. In addition, we compiled a third cohort of TNBC, extracted across 24 different breast cancer cohorts of the Lehmann dataset [26] including 228 tumours of the Karn579 dataset. Applying our previously established DART algorithm [31], for each patient in each cohort a DART score was calculated based on expression of the MaSC gene signature, whereby a high activation score indicated a high similarity of gene expression with the MaSC signature. Heatmap analysis of expression of the 323 genes in the Karn579 dataset ordered from low to high MaSC DART signature tumours (Additional file 13B) confirmed that those tumour profiles with the highest signature scores visibly showed higher average expression of the 323 genes and vice versa. By splitting the Karn579 cohort into those patients with the top tertile DART activation score compared to the remaining samples (Figure 6A), the prognostic value of high MaSC for recurrence-free survival (RFS) was highly significant in TNBC when analysed as a categorical variable (hazard ratio (HR) = 2.165; confidence interval (CI) (1.599 to 2.931); *P* = 5.78e to 07). Many TNBCs with high MaSC expression relapsed in the first 5 years. In the Guy’s107 TNBC cohort we saw the same result for TNBCs with DART scores above the median when considering distant metastasis-free survival (DMFS) (HR = 3.029; CI (1.393 to 6.586); *P* = 0.00517) (Figure 6B) and the extended Lehmann dataset, although not independent of the Karn579 dataset (as noted in the Methods), confirmed this result (HR = 1.52; CI (1.021 to 2.263); *P* = 0.0391) (Figure 6C). Use of the two overlapping data sets (228 tumours in common), demonstrated that the prognostic power of our MaSC gene signature is independent of data set composition and analytical preprocessing of the expression data. Furthermore, analysis of 1,000 random gene lists of the same size as the MaSC signature, and with a similar expression distribution, taken from the Karn579 data illustrated that we would have not observed such a significant *P* value by chance (indicated with a red line in Additional file 14A). Since none of the clinico-pathological features in the 579Karn data showed a significant association with RFS, a multivariate survival analysis was not performed (Additional file 15).

To test the prognostic power of the MaSC gene signature using an orthogonal approach, breast cancer transcriptional profiles were also analysed by deriving a MaSC signature score from the centred, averaged relative expression levels of the MaSC genes for each tumour and including this score in the survival analysis. In agreement with the DART method, the standardised average of the 323 genes was higher in tumours with an overall shorter RFS (Additional file 14B), confirming the relevance of the MaSC biological programme invasion and metastasis in TNBC.

To determine whether the prognostic power of the signature could be extended to ER+ breast cancer, we investigated the activity of the MaSC gene signature in ER+ tumours of three different breast cancer cohorts, namely the NKI295 dataset (226 ER+ tumours/295 samples) [28], the TRANSBIG dataset (134 ER+ tumours/198 samples) [29] and the GSE2990 dataset (149 ER+ tumours/189 samples) [30]. Strikingly, in the ER+ tumours of the NKI295 and the GSE2990 data sets, we observed the opposite effect to that seen in TNBC. ER+ tumours with high MaSC DART activation scores had better DMFS in comparison to the rest (HR = 0.545, CI (0.3374 to 0.8816), *P* = 0.01335 for NKI295; HR = 0.303, CI (0.116 to 0.7913), *P* = 0.01478 for GSE2990) (Figure 6D,E). However, in the TRANSBIG cohort, no association of the MaSC DART activation score could be observed in the ER+ breast cancers (Figure 6F).

### Breast cancer subtype-specific expression of the MaSC signature

**Figure 7:**
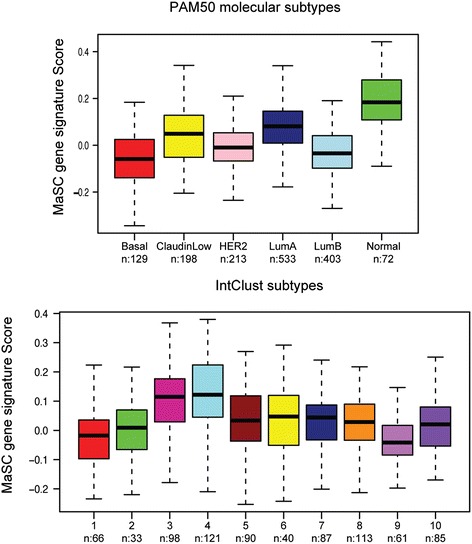
**The MaSC gene signature is enriched in normal-like not claudin-low tumours.** The DART activation score of the MaSC gene signature across breast carcinomas of METABRIC breast cancer cohort. The classification of this cohort into molecularly defined breast cancer subtypes was based on their previously published PAM50 centroid correlation (upper panel) (basal-like in red, HER2 in pink, luminal A in dark blue, luminal B in light blue, and normal-like in green) and across the 10 previously defined IntClust subgroups (lower panel). Number of tumours is shown underneath each subtype. The DART activation score of the MaSC gene signature is shown on the y-axis in both plots. DART, Denoising Algorithm based on Relevance network Topology; HER2, human epidermal growth factor receptor 2; MaSC, mammary stem cell.

Next, we asked whether our MaSC gene signature was associated with a specific subtype across all breast cancers and within TNBCs. We made use of the comprehensive METABRIC breast cancer data set and interrogated which of the PAM50 and IntClust subtypes were enriched for tumours with a high MaSC signature DART activation score. Interestingly, tumours with a high MaSC signature were enriched in the normal-like subtype (Figure 7A), followed by the claudin-low and luminal A subgroup. With the IntClust classification, an enrichment of MaSC signature high tumours was observed in IntClust3 and IntClust4 (Figure 7B). These clusters encompass relatively genomically stable tumours and mainly include luminal A tumours, although IntClust4 also includes subsets of HER2 and basal-like tumours as well as the normal-like group, supporting the PAM50 analysis. Tumours of the IntClust3 and 4 subtypes have been associated with relatively good prognoses [5], in agreement with our results in the ER+ data sets (Figure 6D,E).

**Figure 8:**
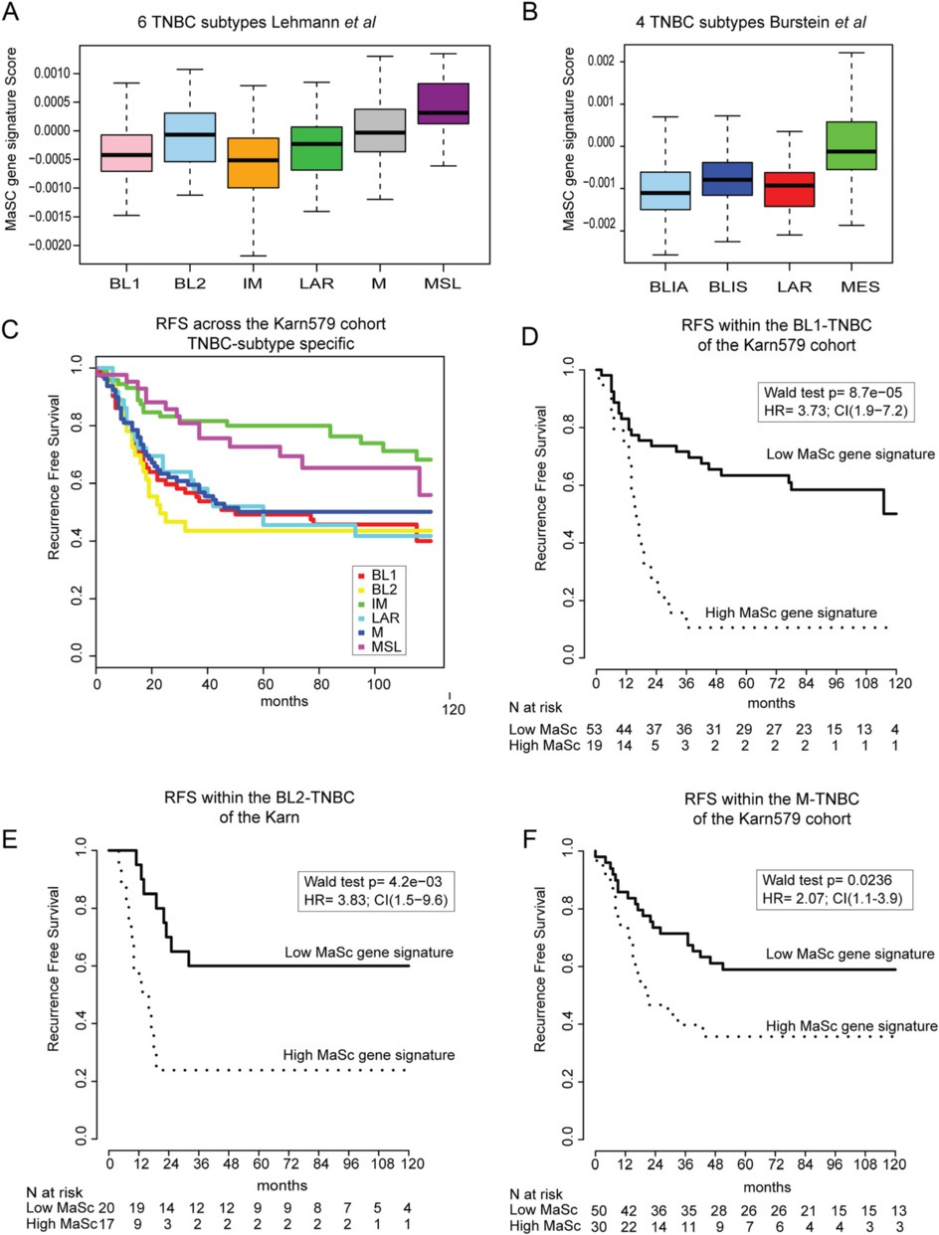
**The MaSC gene signature has prognostic power in specific TNBC subtypes.** The DART activation score of the MaSC gene signature across the Karn579 cohort is shown using **(A)** the Lehmann TNBC subtype classification [26]. TNBC subtypes are represented pink for basal-like 1 (BL1), blue basal-like 2 (BL2), orange immunomodulatory (IM), grey mesenchymal (M), magenta mesenchymal stem-like (MSL), green luminal androgen receptor (LAR), and **(B)** centroid classification of TNBC subtypes defined by Burstein and colleagues [34], namely the ‘luminal androgen receptor (LAR)’, ‘mesenchymal (MES)’, ‘basal-like immune-suppressed (BLIS)’, and ‘basal-like immune activated (BLIA)’ groups. MaSC DART scores are shown in the y-axis. **(C)** Kaplan-Meier analysis of the Karn cohort stratified according to the six TNBC subtypes defined by Lehmann and colleagues [33]. Kaplan-Meier analysis of event-free survival with follow-up information is shown. **(D)** BL1, **(E)** BL2 and **(F)** M classified TNBCs of the Karn579 data were analysed separately and stratified according to the highest tertile of their MaSC DART score activity. DART, Denoising Algorithm based on Relevance network Topology; MaSC, mammary stem cells; TNBC, triple-negative breast cancer.

Finally, we investigated the activation of MaSC gene signature across TNBC subgroups by subtyping the Karn579 cohort with the four TNBC subtypes recently established by Burstein and colleagues [34] namely luminal-AR (LAR), mesenchymal (MES), basal-like immune-suppressed (BLIS), and basal-like immune-activated (BLIA); and the six TNBC subtypes identified by Lehmann and colleagues [33], namely two basal-like (BL1 and BL2), an immunomodulatory (IM), a mesenchymal (M), a mesenchymal stem-like (MSL), and a luminal androgen receptor (LAR) subtype. The tumours with the highest MaSC activation scores were found in the MES subtype in the Burstein classification. In the Lehmann classification the findings were less distinct, with enrichment in the MSL subtype as well as in BL2 and M tumours (Figure 8A,B). To test whether our MaSC can equally stratify these TNBC subtypes with regards to outcome, we first investigated the RFS for each of the five TNBC Lehmann subtypes in the Karn579 cohort (Figure 8C), showing that the MSL and IM had overall better prognosis than the other subtypes. Then we dichotomised each TNBC subtype with our MaSC signature DART score as described above. Survival analysis demonstrated that indeed the BL1, BL2 and M TNBC subtypes could be further stratified by the MaSC signature into those tumours that relapsed within the first 3 years and those with a longer latency period (Figure 8C-F). Taken together, our results demonstrate that we have identified a set of genes that captures a specific stem cell programme that holds biological and clinical (prognostic) information in breast cancer.

## Discussion

TNBC, as a whole, has a poor prognosis and unlike ER+ and HER2+ tumours currently lacks targeted therapies, leaving systemic chemotherapy as the only adjuvant treatment option. These immunohistochemically defined breast carcinomas comprise a histologically, molecularly and clinically highly heterogeneous group of tumours, with some patients having low long-term recurrence rates and responding well to chemotherapy [55]. Thus, there exists a clinical need to stratify patients to ensure the most appropriate treatment is administered. One approach to identification of high-risk disease subgroups in breast cancer is prognostication based on gene expression profiling of primary tumours [56]. Given large tumour cohorts, clinical outcome data and whole transcriptome expression profiles of tumours, it is possible to identify sets of genes whose expression has prognostic value. In ER+ disease, these have typically been genes associated with proliferation [57]. Recent studies have shown that the expression of immune-response genes [58], a metastasis regulator metagene [59] or a chromosomal instability metagene [60] may represent potential prognostic markers for TNBC.

Here, we have used our experience in separation of mammary epithelial cell subsets to isolate a highly purified population of MaSCs and derive a gene signature based on comparison to differentiated MYOs as well as to luminal ER+ and luminal ER- cells. Remarkably, the *a priori*-defined MaSC gene signature was able to provide prognostic information when applied to gene expression profiles of human breast cancers that had undergone no purification protocols or microdissection of tumour tissue. Therefore, the biology of normal MaSCs, as reflected in their gene expression profiles, is highly relevant for understanding the drivers of aggressive disease in TNBC. The gene signature was able to identify TNBC patients with a particularly poor prognosis (especially within the recently identified BL1, BL2 and M subtypes) and who thus might benefit from a more aggressive therapy regime or potential enrolment on to clinical trials of new (targeted) therapies. Clearly, however, further extensive evaluation and refinement of the genes encompassed in this list to maximise its power and general applicability is required before it could be considered usable as a clinical tool.

In contrast to the TNBC data, in two data sets ER+ tumours with a high MaSC activation score had a better prognosis, rather than a worse one. The difference in behaviour of the MaSC gene signature in these tumour types is striking, but the reason for it remains unclear. When all breast cancers in the METABRIC cohort were considered, the MaSC signature was enriched in good prognosis molecular profile tumour subsets (normal-like and Integrative Cluster 3 and 4 tumours), supporting the DART activation score analysis of the ER+ tumour data sets. Moreover, the classification with PAM50 and IntClust demonstrated that MaSC signature is not dividing tumours histologically classified as TNBC into those which are molecularly classified as basal-like and those which are non-basal and have a better prognosis. Notably, the identification of breast cancer stem cells has been based on work primarily from TNBC [53] with little success in identifying ER+ tumour stem cells that will generate a tumour upon xenotransplantation, currently considered the gold standard assay [9]. However, ER+ disease can recur more than 15 years after apparently successful initial treatment [61], which indicates ER+ tumours do possess a cancer stem cell population but one which is quiescent and indolent, in contrast to the aggressive and highly transplantable cancer stem cells that are found in TNBC. This provides one possible explanation why the activity of the MaSC signature identified tumours with opposite RFS in different breast cancer subtypes - cells with features in common with normal stem cells may be the most aggressive in TNBC but, in the short term, the most indolent in ER+ disease (with the potential to become aggressive in the long term). Further extensive analysis is warranted to dissect the differences of intrinsic and environmental factors that will ultimately influence the stem cell behaviour and their association with disease recurrence.

Analysis of the functional role of individual genes within the MaSC signature may shed light on the underlying biology of metastatic disease in TNBC tumours as well as identifying novel therapeutic targets. Of the 15 genes expressed in the MaSC signature as well as in two or more adult mammary stem cell gene signatures from other studies, four (*Cdh13*, *Col17a1*, *Dst* and *Jam2*) are associated with cell junctions and either cell-cell or cell-matrix adhesion and five (*Krt15*, *Mical3*, *Myh11*, *Ppp1r14a* and *Tns1*) are associated with the cytoskeleton or its regulation. Furthermore, *Col17a1* is a hemidesmosome component and therefore linked to the keratin cytoskeleton, *Dst* also has a role in cytoskeletal organisation and *Tns1* is associated with focal adhesions [62]. The regulation of the cytoskeleton by adhesion to other cells and to the matrix therefore seems to be a key component to MaSC biology. Also of interest in the 15 recurrent genes is *Epas1*, which encodes hypoxia inducible factor 2α. Notably, hypoxia in tumours is thought to be an inducer of EMT and cancer stem cell-like activity [63]. Another recurrent theme in regulation of epithelial stem cells is the role of Wnt signalling [64]. For instance, Wnt pathway activation is required to maintain stem cell self-renewal in cultured mammary epithelial cells [65] and Wnt signalling was found to be suppressed in MaSCs after pregnancy [66], consistent with our MaSC gene signature (from virgin animals) being enriched for Wnt pathway-associated genes.

The MaSC signature is of a population with mixed mesenchymal/epithelial features (as supported by the single cell analysis), which would not necessarily be expected to be most strongly enriched in tumours with a pure EMT signature. The mixed nature of the signature also likely explains the small overlap with the signature of Lim and colleagues [10]. There was an overlap of only a single gene with the stem cell signature reported by Stingl and colleagues [12]. However, Stingl and colleagues reported only four genes as upregulated >2 fold in stem cells compared to both MYOs and luminal progenitors (Ma-CFCs, to use their terminology), and the most highly upregulated of these genes was expressed only 2.4 fold higher in the stem cells compared to the other populations. Given these numbers, the single gene overlap (*Fabp4*) is a statistically significant event (Additional file 11). Indeed, *Fabp4* is one of fifteen genes identified in multiple studies including this one as mammary stem cell-specific.

Previous identification of mesenchymal gene expression within the basal population as a whole, encompassing both the myoepithelial and MaSC population, has been largely explained by the contractile role of mature MYOs [9,53,54]. Direct comparison between the MaSC and myoepithelial populations showed that MaSCs express higher levels of some mesenchymal- and epithelial-associated genes such as *Vim* and *Zeb2* (mesenchymal) and *Krt15* (epithelial). We have previously demonstrated by qrtPCR that MaSCs also express *Krt14* approximately 1.5 fold higher than MYOs [37], although the differential is not sufficient to be classed as significantly upregulated in MaSCs in the microarray analysis presented here. Single cell gene expression analysis identified individual MaSCs that either co-expressed *Vim* and *Krt15*, expressed *Vim* or *Krt15* alone or were negative for both. However, immunofluorescent staining of flow-sorted MaSCs showed that, at the protein level, the majority of MaSCs were positive for both Vim and K15. These findings confirmed co-expression of mesenchymal- and epithelial-associated genes in individual MaSCs, although with a transcriptional heterogeneity not necessarily reflected at the protein level. We speculate that this is due to differences in mRNA and protein stability, although confirming this at single cell resolution will be technically challenging. Although a feature of the normal biology of basal mammary epithelial cells, the expression of mesenchymal genes in breast cancer (EMT) has been linked both to aggressive, metastatic tumour progression and the acquisition of stem cell traits in both malignant and normal cells [9,49,50]. However, breast cancers with abundant mesenchymal features (metaplastic spindle cell carcinomas) are rare (approximately 1% of tumours) and fewer than 7% of high-grade breast cancers contain occasional mesenchymal-like cells [3,67,68]. As our findings show, MaSCs express mesenchymal-associated genes, and ‘EMT’ signatures in tumours may in fact reflect the expression of a stem cell-like biological programme by tumour cells rather than a histologically apparent EMT.

We have derived our stem cell signature from basal stem cells defined operationally by their high transplant potential in a cleared mammary fat pad [69]. However, controversy exists as to whether the adult mammary epithelium has a single basal stem cell population that maintains both luminal and basal layers [70] or two distinct stem cell compartments, one basal and one luminal [71]. Evidence for a separate luminal stem cell population has been derived from lineage tracing experiments, as luminal cells have poor transplantation potential [71], although we have shown they can repopulate a cleared fat pad [13]. A recent study has once again provided support for the existence of a common multipotent basal mammary stem cell population defined by the expression of the protein C receptor (Procr^+^) [47]. However, *Procr* expression was not enriched in our MaSC population relative to MYOs in the microarrays. We have defined by transplantation assays, both here (Figure 1) and previously [13], MaSCs as being most highly enriched in the top 5% of basal cells that most strongly express CD49f. In contrast, Procr^+^ cells are found throughout the basal CD24+ CD29+ population, not just in the cells that most strongly express CD29 [47]. qrtPCR analysis of *Procr* expression in the mammary epithelial cell subpopulations confirmed that *Procr* was expressed at similar levels in both MaSCs and MYOs as well as at slightly lower levels in luminal ER+ cells. A full gene expression profile of the Procr^+^ basal cells has not been established [47] so this cannot be used for comparison with the MaSC signature, however, Procr^+^ cells were reported as being enriched for expression of 14 EMT markers. Two of these (*Vim* and *Zeb2*) are expressed in MaSCs.

Both Procr^+^ cells and MaSCs are strongly enriched for mammary fat pad repopulation potential compared to control populations (Procr^-^ basal cells and MYOs, respectively). The transplantation potential of the Procr^+^ basal population may be a result of the activity only of those cells which fall into the top 5% of CD29-expressing basal cells. Furthermore, the transplantation potential of Procr^+^ cells was defined using transplantation with Matrigel [47]; we do not use Matrigel in our transplantation assays. Matrigel is known to improve transplantation potential [72]. It may be that Matrigel enhances the potential of those Procr^+^ cells which fall outside the top 5% of CD29-expressing basal cells. Notably, stem cell frequency of the Procr^+^ basal population was calculated at one in twelve when transplanted with Matrigel [47]; we have previously achieved nine outgrowths from thirty-four transplants when CD24^+/Low^ Sca-1^−^ CD49f^High^ (top 5%) c-Kit^−^ cells were transplanted as single cells without Matrigel [13]. In our hands, at least, *Procr* expression and transplantation potential only partially overlap but understanding the detailed relationship between Procr^+^ basal cells and CD24^+^ CD49f^High^ MaSCs will require extensive lineage tracking, flow sorting and transplantation studies. Nevertheless, the exact relationship between these cell types does not affect our findings that the gene expression signature of the cells we have defined as MaSCs is a strong predictor of outcome in TNBC and, therefore, defines a set of genes that includes some that must be drivers of aggressive behaviour in this tumour subtype.

The basal cell population we have profiled has been selected for its potent transplantation ability [69]. The early events that occur following injection of single mammary basal stem cells into a cleared fat pad are obscure, however, cells that survive this process and can form outgrowths must, by definition, have the ability to survive being reduced to single cells and introduced to a new growth site at low density (indeed, in some experiments even as single cells) [13,73] and then be able to invade and remodel the surrounding environment, forming a new tissue. The parallels with cells that can initiate metastatic dissemination are clear, although not exact, and we speculate that this underlies the strong association between the MaSC gene signature (or, the ‘transplantable basal stem cell gene signature’) and TNBC with high metastatic potential.

## Conclusions

We have tested the hypothesis that genes associated with normal mouse mammary stem cells would have prognostic power in human breast cancer, and we have found that this is indeed the case. Our findings suggest that, as tumour gene expression profiling is based on whole tumour sampling, invasive stem cell-like potential is not limited to a small subset of cells in aggressive TNBC. Furthermore, we have highlighted overlaps between our data set and those of other workers to show that the regulation of cytoskeletal function is a key aspect of MaSC biology. Finally, we have demonstrated that MaSCs have a dual epithelial-mesenchymal identity. Our findings will not only advance our understanding of the molecular regulation of MaSC biology and relationship between the biology of MaSCs and of aggressive, poor prognosis TNBC but also have the potential to inform clinical management of breast cancer, particularly triple-negative disease.

## Additional files

Additional file 1:
**Purification of mouse mammary epithelial cells. (A)** Relationship between mammary epithelial population definitions shown as sets. The size of each set as shown is not proportional to the size of the population within the mammary gland. MaSCs, mammary stem cells. MYOs, myoepithelial cells. **(B)** Full gating cascade of mouse mammary cell preparations from initial scatter plots to gating of total epithelium, as previously defined [18]. **(C)** APC vs FITC and FITC vs PE-Cy5 scatter plots of mammary cells stained with DAPI only to demonstrate gating based on unstained controls. Additional file 2:
**Primer sequences.** TAQMAN Assays-on-Demand references; primer sequences for in-house Sybr Green PCR assays; primer sequences for single cell amplification. Additional file 3:
**Single cell analysis. (A)** Demonstration of linear amplification of exogenous spike controls over two rounds of PCR. Amplification of the three spikes proved to be linear in all samples following PCR 1 and PCR 2 with a non-significant p-value of 1 achieved for all seven cells across both PCR reactions using the Runs test of linearity. This shows that amplification across three molecular values, with a 10-fold dilution between each gene, does not differ significantly from linearity. An analysis of covariance method was used to test if the slope of the line, representing amplification across the three spike levels, varied significantly between independent single cell samples. Following PCR 1 there was no significant difference in the amplification slope between the seven samples (p = 1). Following pair wise comparison of amplification slopes after PCR 2, a significant difference was found between cell 4 and cell 7 (p = 0.049), and cell 6 and cell 7 (p = 0.028). This suggests that a small level of variation is inherent with the second round of PCR amplification. **(B)** Single cell cDNA amplification on sixteen single and sixteen groups of ten CommaD cells. qrtPCR for seven genes (Gapdh, Ubc, Jag1, Jag2, Wnt4, Wnt5a and Wnt10a) was performed on the single cell and 10-cell samples and on unamplified cDNA collected from the bulk population. The mean of sixteen single cell expression levels for each gene was compared to the mean of expression levels from the sixteen 10-cell samples (left hand plot). The mean single cell amplified expression levels for the seven genes were also compared to unamplified cDNA (right hand plot). Additional file 4:
**Clinico-pathological features of the Lehmann dataset** **[** 26 **].**
Additional file 5:
**Examples of staining of single mammary cells. (A)** Single mammary cells single stained for K14, K18 and ERα. **(B)** Single mammary cells (one K14+/K18- MaSC; two (weak) K14+/K18+ MaSCs; one K14-/K18+ luminal cell) sorted on to slides and double stained for expression of K14 and K18, and counterstained with DAPI. Bar = 10 uM. Additional file 6:
**Batch analysis of raw microarray data and** ***Procr*** **gene expression data. (A)** Boxplot showing distribution of gene expression in raw data sets from previous analyses [16] and the new MYO and MaSC data sets. No evidence of a batch effect is seen. **(B)** qrtPCR analysis of *Procr* gene expression in mammary epithelial subpopulations. Data expressed as mean fold expression (±95% confidence intervals) over comparator population (luminal ER- cells) in three independent isolates of each cell population. Statistical significance was determined according to [74]. ^*^ *P* <0.05, ^**^ *P* <0.01, N.S. not significant. Additional file 7:
**Fold expression of 323 MaSC-specific genes over myoepithelial and luminal cells ordered by fold expression relative to myoepithelial (upper table) and luminal (lower table) populations.**
Additional file 8:
**Gene Ontology (GO) and Pathway analysis of MaSC-specific genes.**
Additional file 9:
**Top 100 genes most strongly downregulated in MaSCs compared to MYOs.**
Additional file 10:
**GO analysis of genes downregulated in MaSCs.**
Additional file 11:
**Overlap of MaSC-specific genes with published stem cell gene signatures** **[** 15 **-** 17 **,** 40 **,** 41 **].**
Additional file 12:
**Table of Pearson correlations for gene expression in MaSCs analysed at single cell resolution.**
Additional file 13:
**Staining of basal cells for epithelial and mesenchymal markers and heat map of MaSC signature gene expression in Karn579 tumours ordered by DART score. (A)** Immunohistochemical staining of two representative regions of mammary epithelium from normal mouse mammary gland for an epithelial (K14) and two mesenchymal (SMA and Vimentin) antigens. Region 1 is an oblique section through the edge of a duct. Bars = 10 um. Note strong staining of basal layer for K14 and SMA, and weaker, punctate staining for Vimentin in a subset of cells (Vimentin-positive cells within the mammary epithelium indicated with an asterisk; the grey dashed line indicates the boundary between the mammary epithelium and the mammary stroma, which includes Vimentin-positive stromal cells). **(B)** Heat map of expression of the 323 MaSC signature genes in the Karn579 dataset ordered left to right from high DART score tumours to low DART score tumours. Red indicates high expression, green low expression. Additional file 14:
**The MaSC gene signature performs better than random gene lists. (A)** Predictive power of 1,000 random gene sets compared to the MaSC gene signature. The frequency of obtained *P* values is shown in the y-axis, while the -log 10 *P* values are listed on the x-axis. The black line indicates the *P* values of the 1,000 gene sets; the *P* value of the MaSC signature is indicated by the red line. **(B)** Averaged MaSC signature scores in tumours are correlated with patient outcome, supporting the prognostic power of the DART score. Additional file 15:
**Univariate analysis of prognostic factors in the Karn dataset.**

## Abbreviations

BL1/2: basal-like 1/2
BLIA: basal-like immune-activated
BLIS: basal-like immune-suppressed
CI: confidence interval
DART: Denoising Algorithm based on Relevance network Topology
DMFS: distant metastasis-free survival
EMT: epithelial-mesenchymal transition
*Esr1*: ER, estrogen receptor α (gene and protein)
FCS: foetal calf serum
FDR: false discovery rate
GO: Gene Ontology
HER2: human epidermal growth factor receptor 2
HR: hazard ratio
*Krt14*: K14, keratin 14 (gene and protein)
IFF: immunofluorescence buffer
IM: immunomodulatory
K15: keratin 15
*Krt18*: K18, keratin 18 (gene and protein)
LAR: luminal androgen receptor
MaSC: mammary stem cell
MBE: mammary bud epithelium
MBM: mammary bud mesenchyme
MES: mesenchymal
MSL: mesenchymal stem-like
MYO: myoepithelial cell
PBS: phosphate-buffered saline
PR: progesterone receptor
qrtPCR: quantitative real-time rtPCR
RMA: robust microarray analysis
RFS: recurrence-free survival
RT: room temperature
SAM: significance analysis of microarrays
SMA: smooth muscle actin
TBS: Tris-buffered saline
TdT: terminal deoxynucleotidyl transferase
TNBC: triple-negative breast cancer
Vim: vimentin

## References

[1] Lakhani SR, Ellis IO, Schnitt SJ, Tan PH, Van De Vijver MJ (2012). WHO classification of tumors of the breast.

[2] Carey LA, Dees EC, Sawyer L, Gatti L, Moore DT, Collichio F (2007). The triple negative paradox: primary tumor chemosensitivity of breast cancer subtypes. Clin Cancer Res..

[3] Reis-Filho JS, Milanezi F, Steele D, Savage K, Simpson PT, Nesland JM (2006). Metaplastic breast carcinomas are basal-like tumours. Histopathology..

[4] Weigelt B, Reis-Filho JS (2009). Histological and molecular types of breast cancer: is there a unifying taxonomy?. Nat Rev Clin Oncol..

[5] Curtis C, Shah SP, Chin SF, Turashvili G, Rueda OM, Dunning MJ (2012). The genomic and transcriptomic architecture of 2,000 breast tumours reveals novel subgroups. Nature..

[6] Sorlie T, Tibshirani R, Parker J, Hastie T, Marron JS, Nobel A (2003). Repeated observation of breast tumor subtypes in independent gene expression data sets. Proc Natl Acad Sci U S A..

[7] van’t Veer LJ, Dai H, van de Vijver MJ, He YD, Hart AA, Mao M (2002). Gene expression profiling predicts clinical outcome of breast cancer. Nature..

[8] Liedtke C, Mazouni C, Hess KR, Andre F, Tordai A, Mejia JA (2008). Response to neoadjuvant therapy and long-term survival in patients with triple-negative breast cancer. J Clin Oncol..

[9] Smalley M, Piggott L, Clarkson R (2013). Breast cancer stem cells: obstacles to therapy. Cancer Lett..

[10] Lim E, Wu D, Pal B, Bouras T, Asselin-Labat ML, Vaillant F (2010). Transcriptome analyses of mouse and human mammary cell subpopulations reveals multiple conserved genes and pathways. Breast Cancer Res..

[11] Pece S, Tosoni D, Confalonieri S, Mazzarol G, Vecchi M, Ronzoni S (2010). Biological and molecular heterogeneity of breast cancers correlates with their cancer stem cell content. Cell..

[12] Stingl J, Eirew P, Ricketson I, Shackleton M, Vaillant F, Choi D (2006). Purification and unique properties of mammary epithelial stem cells. Nature..

[13] Regan JL, Kendrick H, Magnay FA, Vafaizadeh V, Groner B, Smalley MJ (2012). c-Kit is required for growth and survival of the cells of origin of Brca1-mutation-associated breast cancer. Oncogene..

[14] Sleeman KE, Kendrick H, Robertson D, Isacke CM, Ashworth A, Smalley MJ (2007). Dissociation of estrogen receptor expression and in vivo stem cell activity in the mammary gland. J Cell Biol..

[15] Significance analysis of microarrays. [http://www-stat.stanford.edu/~tibs/SAM/]

[16] Kendrick H, Regan JL, Magnay FA, Grigoriadis A, Mitsopoulos C, Zvelebil M (2008). Transcriptome analysis of mammary epithelial subpopulations identifies novel determinants of lineage commitment and cell fate. BMC Genomics..

[17] Sims D, Bursteinas B, Gao Q, Jain E, MacKay A, Mitsopoulos C, Zvelebil M. ROCK: a breast cancer functional genomics resource. Breast Cancer Res Treat. 2010;124(2):567–72. http://www.ncbi.nlm.nih.gov/pubmed/20563840. 10.1007/s10549-010-0945-5 20563840

[18] Ng A, Bursteinas B, Gao Q, Mollison E, Zvelebil M (2006). pSTIING: a ‘systems’ approach towards integrating signalling pathways, interaction and transcriptional regulatory networks in inflammation and cancer. Nucleic Acids Res..

[19] da Huang W, Sherman BT, Lempicki RA (2009). Systematic and integrative analysis of large gene lists using DAVID bioinformatics resources. Nat Protoc..

[20] da Huang W, Sherman BT, Lempicki RA (2009). Bioinformatics enrichment tools: paths toward the comprehensive functional analysis of large gene lists. Nucleic Acids Res..

[21] VENNY. An interactive tool for comparing lists with Venn Diagrams. [http://bioinfogp.cnb.csic.es/tools/venny/index.html]

[22] Tan DW, Jensen KB, Trotter MW, Connelly JT, Broad S, Watt FM (2013). Single-cell gene expression profiling reveals functional heterogeneity of undifferentiated human epidermal cells. Development..

[23] Deugnier MA, Faraldo MM, Teuliere J, Thiery JP, Medina D, Glukhova MA (2006). Isolation of mouse mammary epithelial progenitor cells with basal characteristics from the Comma-Dbeta cell line. Dev Biol..

[24] Karn T, Pusztai L, Holtrich U, Iwamoto T, Shiang CY, Schmidt M (2011). Homogeneous datasets of triple negative breast cancers enable the identification of novel prognostic and predictive signatures. PLoS One..

[25] de Rinaldis E, Gazinska P, Mera A, Modrusan Z, Fedorowicz GM, Burford B (2013). Integrated genomic analysis of triple-negative breast cancers reveals novel microRNAs associated with clinical and molecular phenotypes and sheds light on the pathways they control. BMC Genomics..

[26] Lehmann BD, Bauer JA, Chen X, Sanders ME, Chakravarthy AB, Shyr Y (2011). Identification of human triple-negative breast cancer subtypes and preclinical models for selection of targeted therapies. J Clin Invest..

[27] Johnson WE, Li C, Rabinovic A (2007). Adjusting batch effects in microarray expression data using empirical Bayes methods. Biostatistics..

[28] van de Vijver MJ, He YD, van’t Veer LJ, Dai H, Hart AA, Voskuil DW (2002). A gene-expression signature as a predictor of survival in breast cancer. N Engl J Med..

[29] Desmedt C, Piette F, Loi S, Wang Y, Lallemand F, Haibe-Kains B (2007). Strong time dependence of the 76-gene prognostic signature for node-negative breast cancer patients in the TRANSBIG multicenter independent validation series. Clin Cancer Res..

[30] Sotiriou C, Wirapati P, Loi S, Harris A, Fox S, Smeds J (2006). Gene expression profiling in breast cancer: understanding the molecular basis of histologic grade to improve prognosis. J Natl Cancer Inst..

[31] Jiao Y, Lawler K, Patel GS, Purushotham A, Jones AF, Grigoriadis A (2011). DART: Denoising Algorithm based on Relevance network Topology improves molecular pathway activity inference. BMC Bioinformatics..

[32] Parker JS, Mullins M, Cheang MC, Leung S, Voduc D, Vickery T (2009). Supervised risk predictor of breast cancer based on intrinsic subtypes. J Clin Oncol..

[33] Chen X, Li J, Gray WH, Lehmann BD, Bauer JA, Shyr Y (2012). TNBCtype: a subtyping tool for triple-negative breast cancer. Cancer Inform..

[34] Burstein MD, Tsimelzon A (2014). Poage GM.

[35] R-project. [http://www.r-project.org/]

[36] Britt KL, Kendrick H, Regan JL, Molyneux G, Magnay FA, Ashworth A (2009). Pregnancy in the mature adult mouse does not alter the proportion of mammary epithelial stem/progenitor cells. Breast Cancer Res..

[37] Molyneux G, Geyer FC, Magnay FA, McCarthy A, Kendrick H, Natrajan R (2010). BRCA1 basal-like breast cancers originate from luminal epithelial progenitors and not from basal stem cells. Cell Stem Cell..

[38] Mansson R, Hultquist A, Luc S, Yang L, Anderson K, Kharazi S (2007). Molecular evidence for hierarchical transcriptional lineage priming in fetal and adult stem cells and multipotent progenitors. Immunity..

[39] Spike BT, Engle DD, Lin JC, Cheung SK, La J, Wahl GM (2012). A mammary stem cell population identified and characterized in late embryogenesis reveals similarities to human breast cancer. Cell Stem Cell..

[40] Wansbury O, Mackay A, Kogata N, Mitsopoulos C, Kendrick H, Davidson K (2011). Transcriptome analysis of embryonic mammary cells reveals insights into mammary lineage establishment. Breast Cancer Res..

[41] Horsley V, Aliprantis AO, Polak L, Glimcher LH, Fuchs E (2008). NFATc1 balances quiescence and proliferation of skin stem cells. Cell..

[42] Shimono Y, Zabala M, Cho RW, Lobo N, Dalerba P, Qian D (2009). Downregulation of miRNA-200c links breast cancer stem cells with normal stem cells. Cell..

[43] Wong DJ, Liu H, Ridky TW, Cassarino D, Segal E, Chang HY (2008). Module map of stem cell genes guides creation of epithelial cancer stem cells. Cell Stem Cell..

[44] Frings O, Augsten M, Tobin NP, Carlson J, Paulsson J, Pena C (2013). Prognostic significance in breast cancer of a gene signature capturing stromal PDGF signaling. Am J Pathol..

[45] Hope KJ, Cellot S, Ting SB, MacRae T, Mayotte N, Iscove NN (2010). An RNAi screen identifies Msi2 and Prox1 as having opposite roles in the regulation of hematopoietic stem cell activity. Cell Stem Cell..

[46] Ernst A, Hofmann S, Ahmadi R, Becker N, Korshunov A, Engel F (2009). Genomic and expression profiling of glioblastoma stem cell-like spheroid cultures identifies novel tumor-relevant genes associated with survival. Clin Cancer Res..

[47] Wang D, Cai C, Dong X, Yu QC, Zhang XO, Yang L (2015). Identification of multipotent mammary stem cells by protein C receptor expression. Nature..

[48] Shan T, Liu W, Kuang S (2013). Fatty acid binding protein 4 expression marks a population of adipocyte progenitors in white and brown adipose tissues. Faseb J..

[49] Iliopoulos D, Hirsch HA, Wang G, Struhl K (2011). Inducible formation of breast cancer stem cells and their dynamic equilibrium with non-stem cancer cells via IL6 secretion. Proc Natl Acad Sci U S A..

[50] Mani SA, Guo W, Liao M-J, Eaton EN, Ayyanan A, Zhou AY (2008). The epithelial-mesenchymal transition generates cells with properties of stem cells. Cell..

[51] Smalley MJ, Titley J, O’Hare MJ (1998). Clonal characterization of mouse mammary luminal epithelial and myoepithelial cells separated by fluorescence-activated cell sorting. In Vitro Cell Dev Biol Anim..

[52] Smalley MJ (1995). Clonal characterisation of mouse mammary luminal epithelial and myoepithelial cells.

[53] Al-Hajj M, Wicha MS, Benito-Hernandez A, Morrison SJ, Clarke MF (2003). Prospective identification of tumorigenic breast cancer cells. Proc Natl Acad Sci U S A..

[54] Nakshatri H, Srour EF, Badve S (2009). Breast cancer stem cells and intrinsic subtypes: controversies rage on. Curr Stem Cell Res Ther..

[55] Haffty BG, Yang Q, Reiss M, Kearney T, Higgins SA, Weidhaas J (2006). Locoregional relapse and distant metastasis in conservatively managed triple negative early-stage breast cancer. J Clin Oncol..

[56] Reis-Filho JS, Pusztai L (2011). Gene expression profiling in breast cancer: classification, prognostication, and prediction. Lancet..

[57] Sotiriou C, Pusztai L (2009). Gene-expression signatures in breast cancer. N Engl J Med..

[58] Irshad S, Grigoriadis A, Lawler K, Ng T, Tutt A (2012). Profiling the immune stromal interface in breast cancer and its potential for clinical impact. Breast Care (Basel)..

[59] Lee U, Frankenberger C, Yun J, Bevilacqua E, Caldas C, Chin SF (2013). A prognostic gene signature for metastasis-free survival of triple negative breast cancer patients. PLoS One..

[60] Al-Ejeh F, Simpson PT, Sanus JM, Klein K, Kalimutho M, Shi W (2014). Meta-analysis of the global gene expression profile of triple-negative breast cancer identifies genes for the prognostication and treatment of aggressive breast cancer. Oncogenesis..

[61] Dent R, Trudeau M, Pritchard KI, Hanna WM, Kahn HK, Sawka CA (2007). Triple-negative breast cancer: clinical features and patterns of recurrence. Clin Cancer Res..

[62] SOURCE Database. [http://smd.stanford.edu/cgi-bin/source/sourceSearch]

[63] Hill RP, Marie-Egyptienne DT, Hedley DW (2009). Cancer stem cells, hypoxia and metastasis. Semin Radiat Oncol..

[64] Wend P, Holland JD, Ziebold U, Birchmeier W (2010). Wnt signaling in stem and cancer stem cells. Semin Cell Dev Biol..

[65] Zeng YA, Nusse R (2010). Wnt proteins are self-renewal factors for mammary stem cells and promote their long-term expansion in culture. Cell Stem Cell..

[66] Meier-Abt F, Milani E, Roloff T, Brinkhaus H, Duss S, Meyer DS (2013). Parity induces differentiation and reduces Wnt/Notch signaling ratio and proliferation potential of basal stem/progenitor cells isolated from mouse mammary epithelium. Breast Cancer Res..

[67] Lee H, Jung SY, Ro JY, Kwon Y, Sohn JH, Park IH (2012). Metaplastic breast cancer: clinicopathological features and its prognosis. J Clin Pathol..

[68] Fulford LG, Easton DF, Reis-Filho JS, Sofronis A, Gillett CE, Lakhani SR (2006). Specific morphological features predictive for the basal phenotype in grade 3 invasive ductal carcinoma of breast. Histopathology..

[69] Smalley MJ, Kendrick H, Sheridan JM, Regan JL, Prater MD, Lindeman GJ (2012). Isolation of mouse mammary epithelial subpopulations: a comparison of leading methods. J Mammary Gland Biol Neoplasia..

[70] Rios AC, Fu NY, Lindeman GJ, Visvader JE (2014). In situ identification of bipotent stem cells in the mammary gland. Nature..

[71] Van Keymeulen A, Rocha AS, Ousset M, Beck B, Bouvencourt G, Rock J (2011). Distinct stem cells contribute to mammary gland development and maintenance. Nature..

[72] Quintana E, Shackleton M, Sabel MS, Fullen DR, Johnson TM, Morrison SJ (2008). Efficient tumour formation by single human melanoma cells. Nature..

[73] Shackleton M, Vaillant F, Simpson KJ, Stingl J, Smyth GK, Asselin-Labat ML (2006). Generation of a functional mammary gland from a single stem cell. Nature..

[74] Cumming G, Fidler F, Vaux DL (2007). Error bars in experimental biology. J Cell Biol..

