# Supplementary material for: Mouse mammary stem cells express prognostic markers for triple-negative breast cancer
Source: Breast Cancer Res. 2015 Mar 4;17(1):31. doi: 10.1186/s13058-015-0539-6 (PMC4381533; doi:10.1186/s13058-015-0539-6)

A

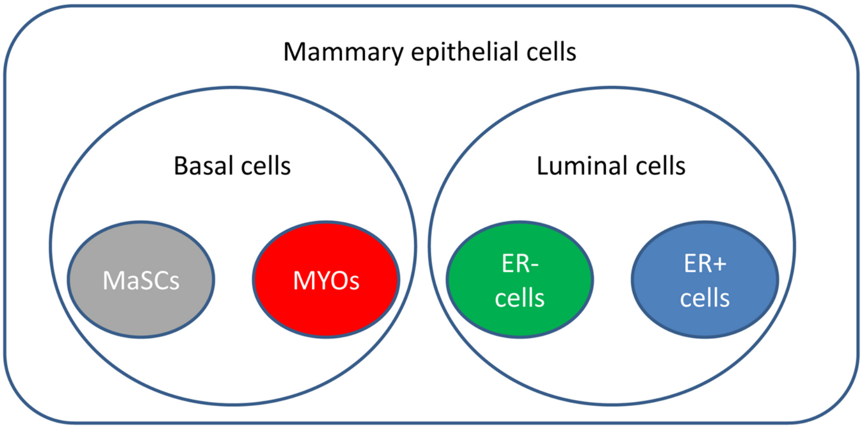

B

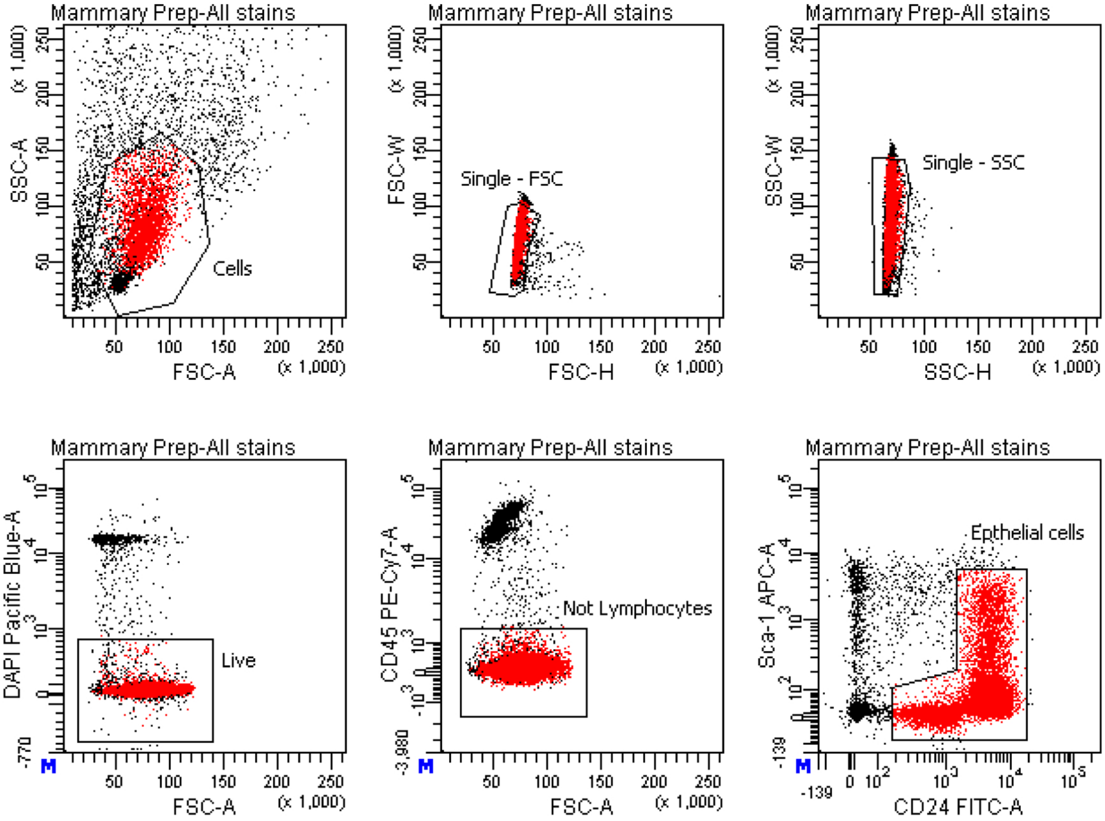

Tube: All stains

Population	#Events	%Parent	%Total
All Events	30,000	####	100.0
Cells	15,451	51.5	51.5
Single - FSC	15,191	98.3	50.6
Single - SSC	14,893	98.0	49.6
Live	14,125	94.8	47.1
Not Lymphocytes	11,970	84.7	39.9
Epthelial cells	10,073	84.2	33.6

C

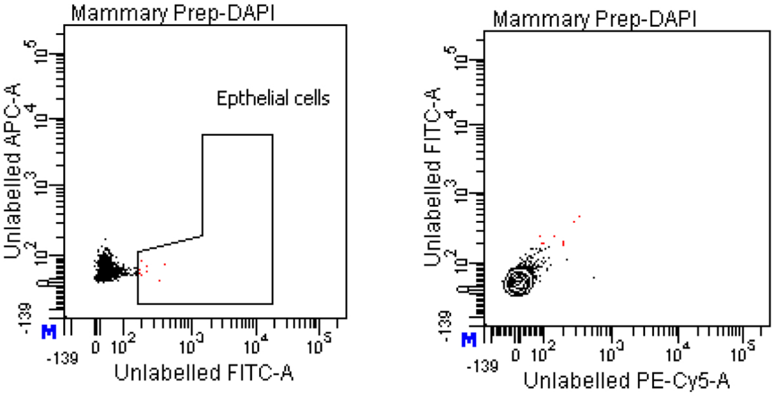

Supplement: Additional file 1: — Purification of mouse mammary epithelial cells. (A) Relationship between mammary epithelial population definitions shown as sets. The size of each set as shown is not proportional to the size of the population within the mammary gland. MaSCs, mammary stem cells. MYOs, myoepithelial cells. (B) Full gating cascade of mouse mammary cell preparations from initial scatter plots to gating of total epithelium, as previously defined [18]. (C) APC vs FITC and FITC vs PE-Cy5 scatter plots of mammary cells stained with DAPI only to demonstrate gating based on unstained controls. [file 13058_2015_539_MOESM1_ESM.pdf]
