# Supplementary material for: Mouse mammary stem cells express prognostic markers for triple-negative breast cancer
Source: Breast Cancer Res. 2015 Mar 4;17(1):31. doi: 10.1186/s13058-015-0539-6 (PMC4381533; doi:10.1186/s13058-015-0539-6)

A

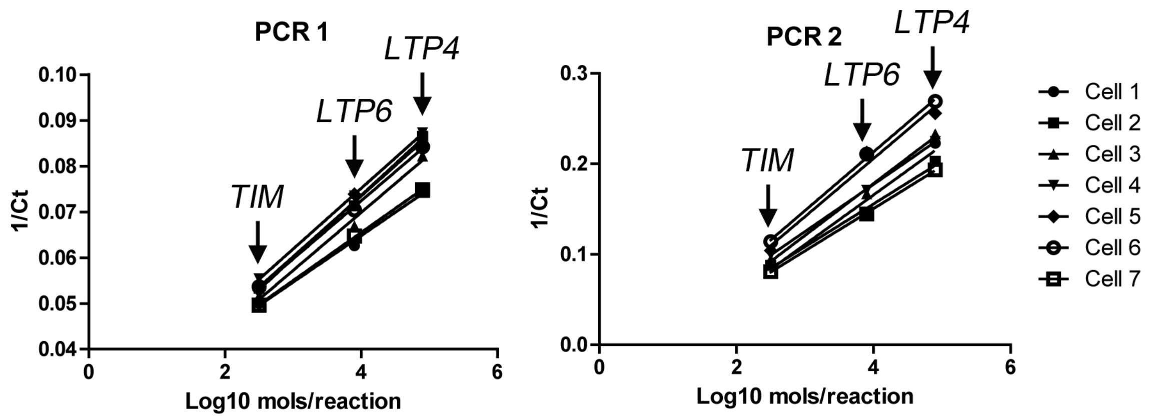

Demonstration of linear amplification of exogenous spike controls over two rounds of PCR. Freshly isolated mammary epithelial cells were single cell sorted directly into thin walled 96 well PCR plates containing the first strand synthesis buffer. This was immediately frozen on dry ice and stored at −20 °C until the full amplification method could be performed. qrtPCR for spike controls LTP4, LTP6 and TIM was carried out in triplicate on the products of PCR 1 and PCR 2 from the single cellamplification of seven myoepithelial cells. The linearity of amplification was tested by converting the picogram quantities of each internal spike control to represent molecular amounts using the following formula X g µl−1 RNA / (transcript length x 340) x 6.022 x 1023. Therefore, LTP4 added at 10−2 pg was present at 8400 molecules, LTP6 added at 10−3 pg was present at 900 molecules, and TIM added at 10−4 pg was present at 90 molecules per reaction. These values were log transformed and plotted against 1/Ct qrtPCR expression levels.

B

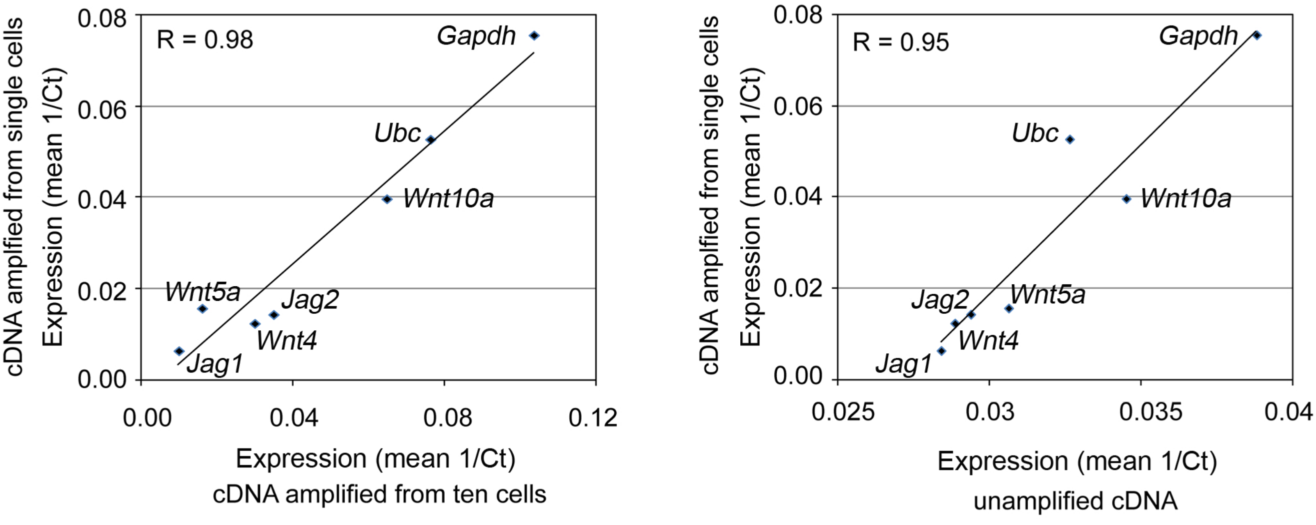

Supplement: Additional file 3: — Single cell analysis. (A) Demonstration of linear amplification of exogenous spike controls over two rounds of PCR. Amplification of the three spikes proved to be linear in all samples following PCR 1 and PCR 2 with a non-significant p-value of 1 achieved for all seven cells across both PCR reactions using the Runs test of linearity. This shows that amplification across three molecular values, with a 10-fold dilution between each gene, does not differ significantly from linearity. An analysis of covariance method was used to test if the slope of the line, representing amplification across the three spike levels, varied significantly between independent single cell samples. Following PCR 1 there was no significant difference in the amplification slope between the seven samples (p = 1). Following pair wise comparison of amplification slopes after PCR 2, a significant difference was found between cell 4 and cell 7 (p = 0.049), and cell 6 and cell 7 (p = 0.028). This suggests that a small level of variation is inherent with the second round of PCR amplification. (B) Single cell cDNA amplification on sixteen single and sixteen groups of ten CommaD cells. qrtPCR for seven genes (Gapdh, Ubc, Jag1, Jag2, Wnt4, Wnt5a and Wnt10a) was performed on the single cell and 10-cell samples and on unamplified cDNA collected from the bulk population. The mean of sixteen single cell expression levels for each gene was compared to the mean of expression levels from the sixteen 10-cell samples (left hand plot). The mean single cell amplified expression levels for the seven genes were also compared to unamplified cDNA (right hand plot). [file 13058_2015_539_MOESM3_ESM.pdf]
