# Supplementary figures and images for: Mouse mammary stem cells express prognostic markers for triple-negative breast cancer
Source: Breast Cancer Res. 2015 Mar 4;17(1):31. doi: 10.1186/s13058-015-0539-6 (PMC4381533; doi:10.1186/s13058-015-0539-6)

A

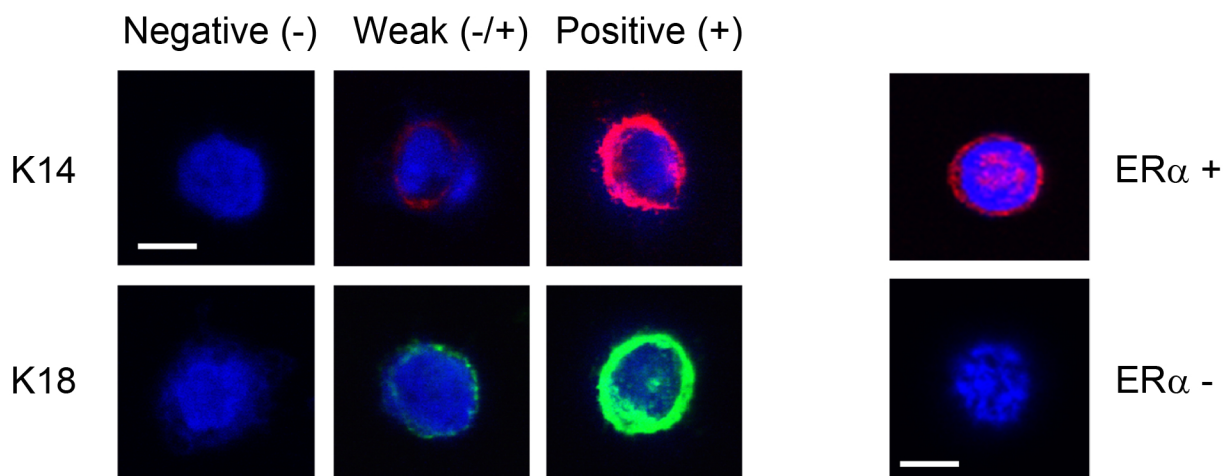

B

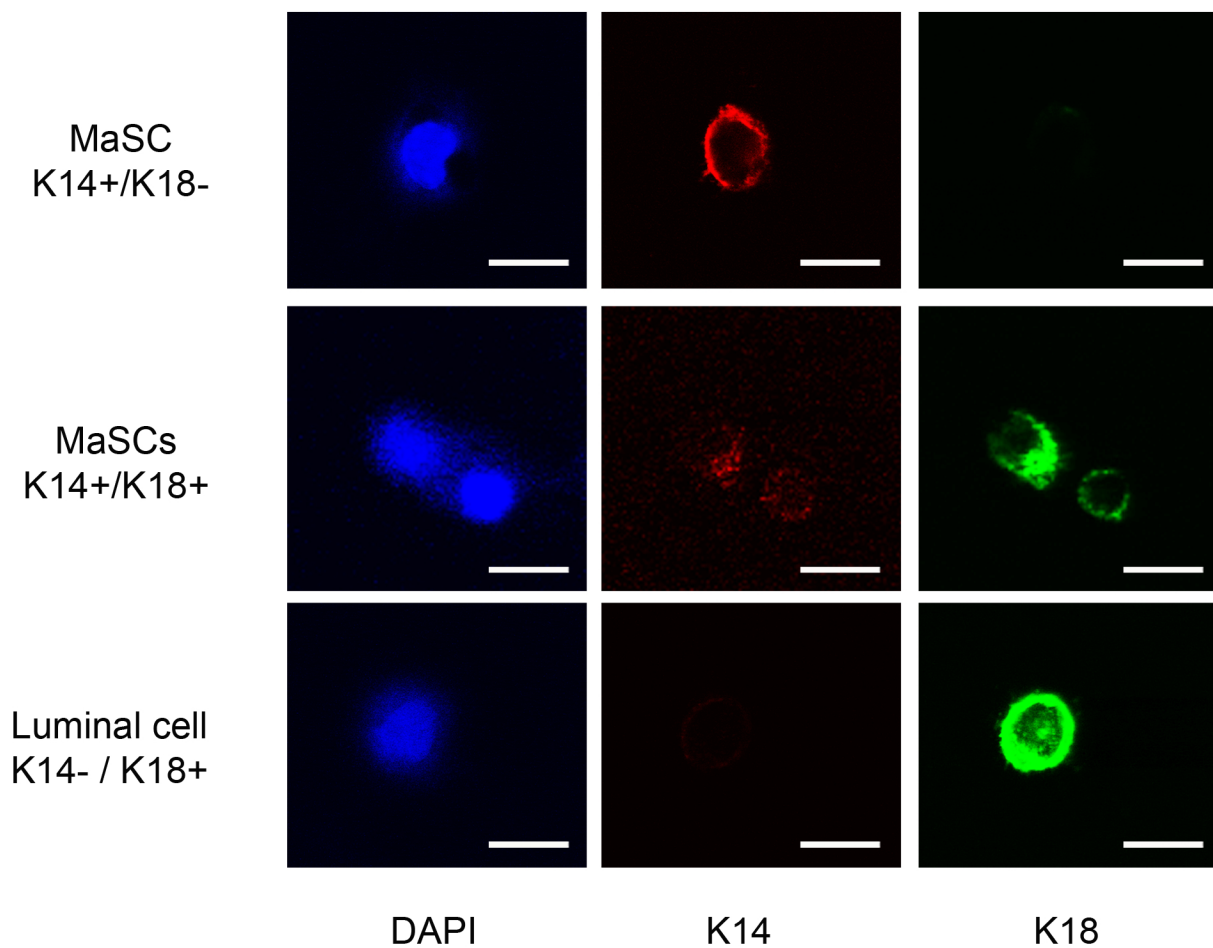

Supplement: Additional file 5: — Examples of staining of single mammary cells. (A) Single mammary cells single stained for K14, K18 and ERα. (B) Single mammary cells (one K14+/K18- MaSC; two (weak) K14+/K18+ MaSCs; one K14-/K18+ luminal cell) sorted on to slides and double stained for expression of K14 and K18, and counterstained with DAPI. Bar = 10 uM. [file 13058_2015_539_MOESM5_ESM.pdf]

A

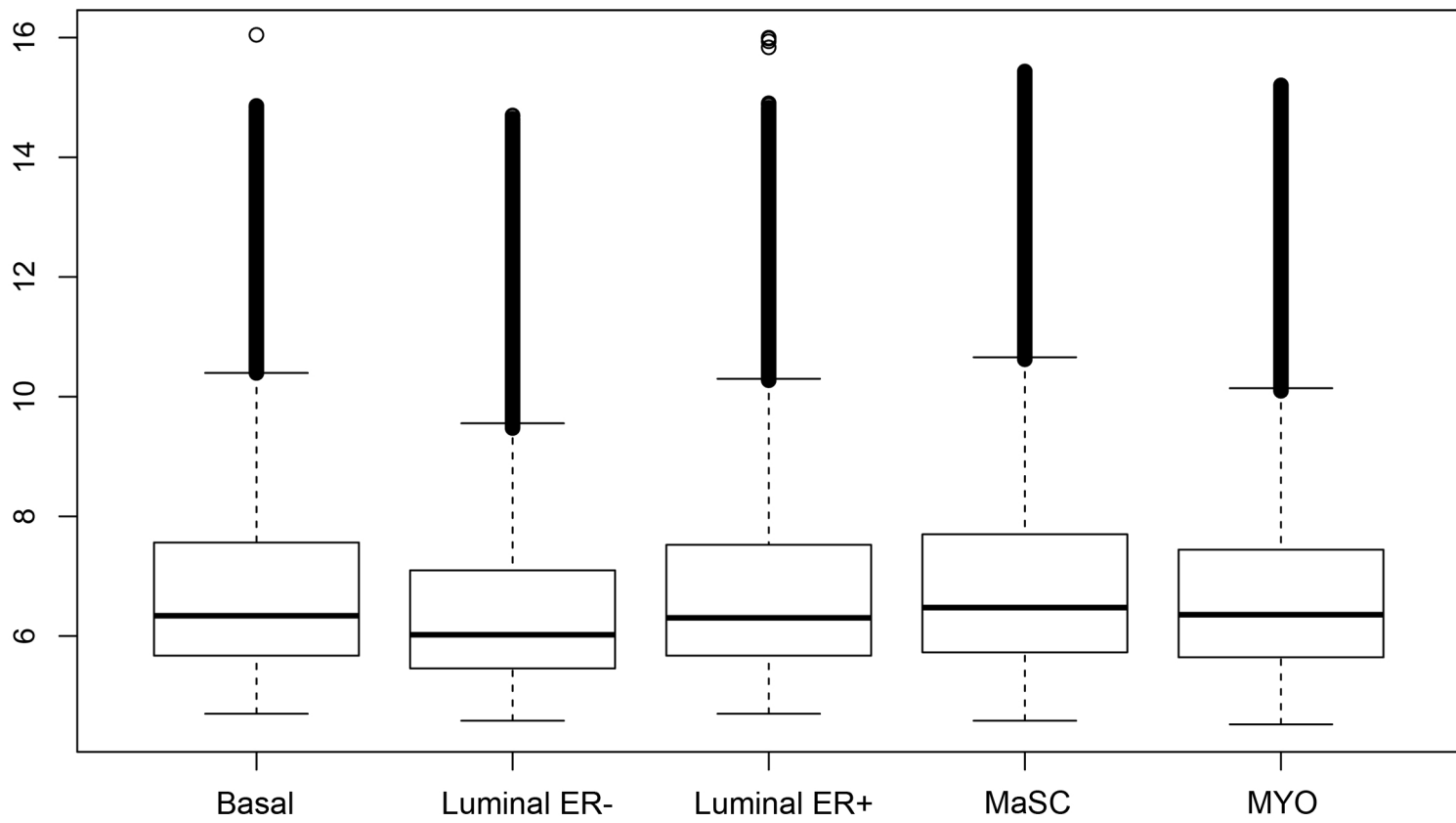

B

*Procr* expression in mammary epithelial cell subpopulations

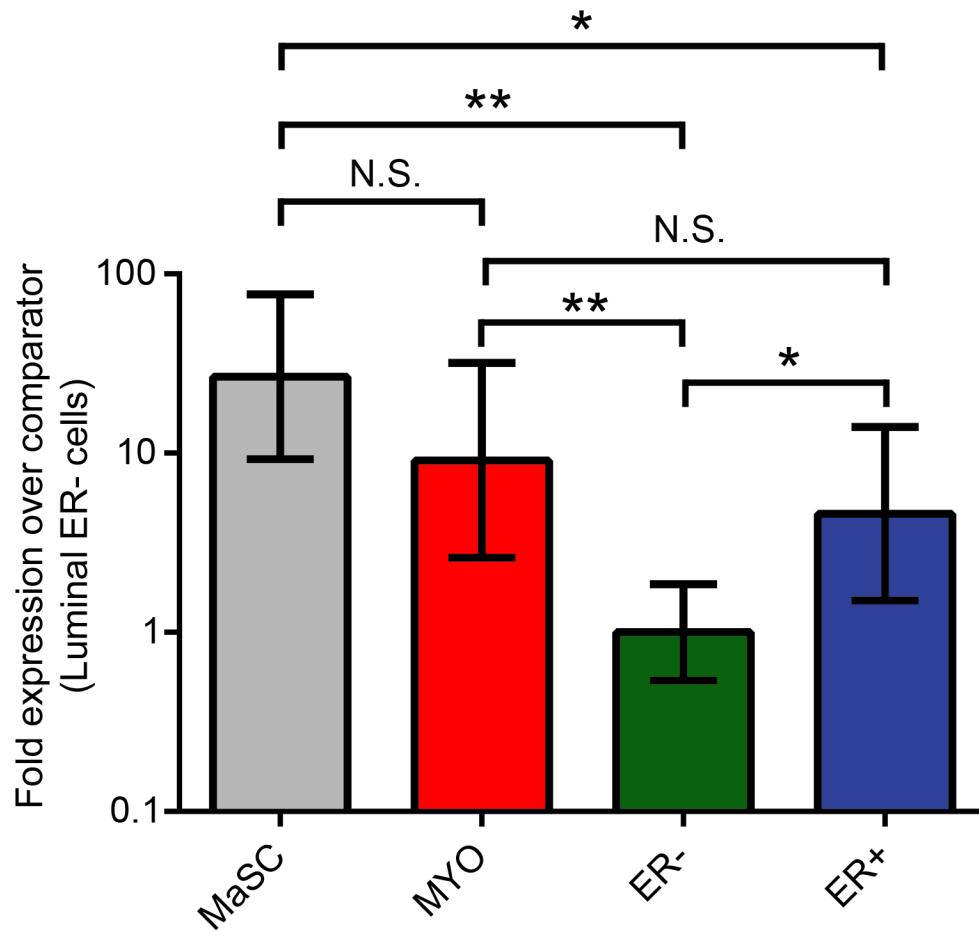

Supplement: Additional file 6: — Batch analysis of raw microarray data and Procr gene expression data. (A) Boxplot showing distribution of gene expression in raw data sets from previous analyses [16] and the new MYO and MaSC data sets. No evidence of a batch effect is seen. (B) qrtPCR analysis of Procr gene expression in mammary epithelial subpopulations. Data expressed as mean fold expression (±95% confidence intervals) over comparator population (luminal ER- cells) in three independent isolates of each cell population. Statistical significance was determined according to [74]. * P <0.05, ** P <0.01, N.S. not significant. [file 13058_2015_539_MOESM6_ESM.pdf]

A

Evaluation of significance for MaSC genesignature  
with random sampling analysis

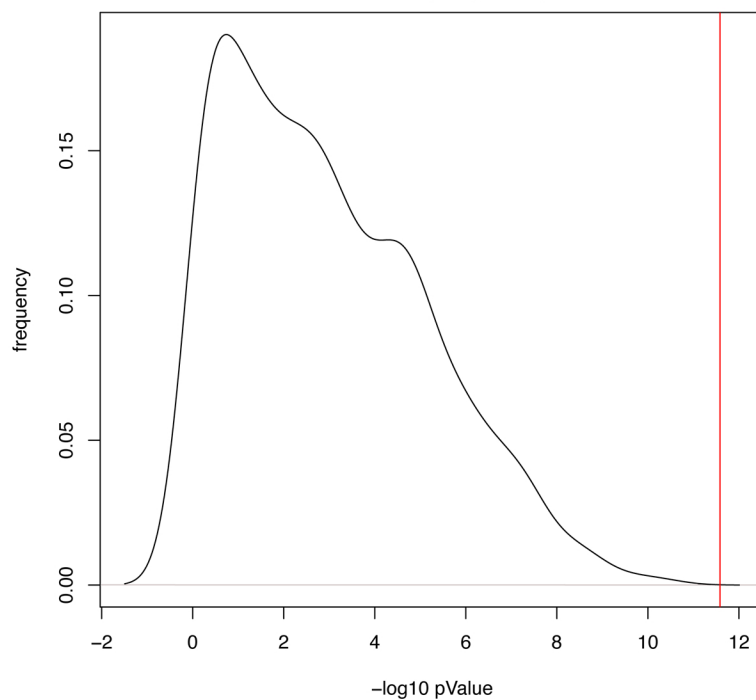

B

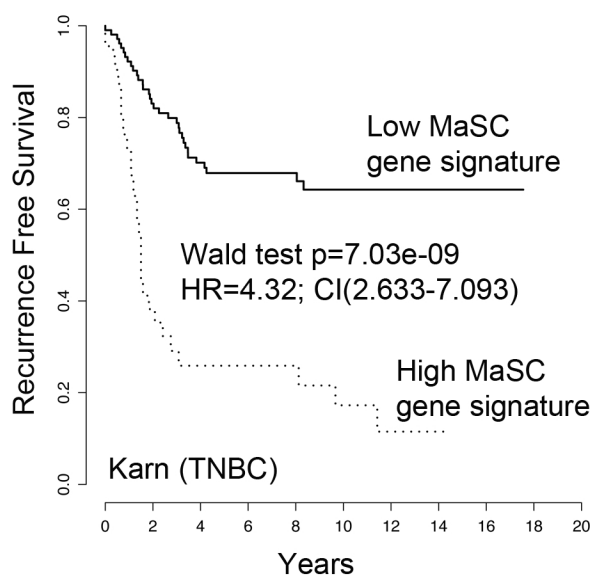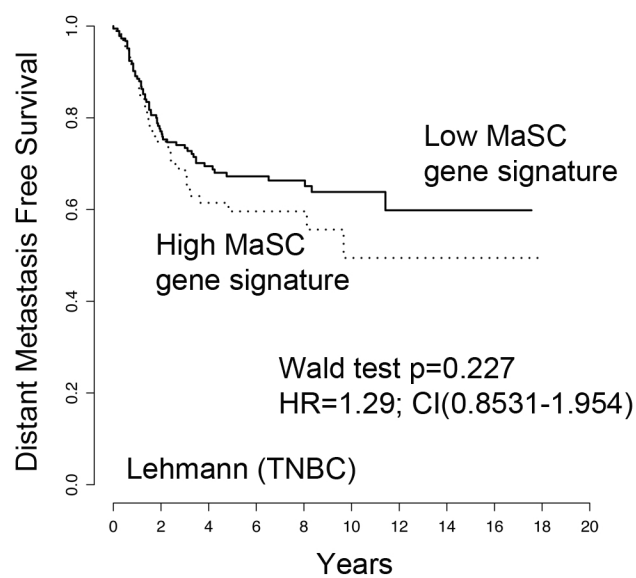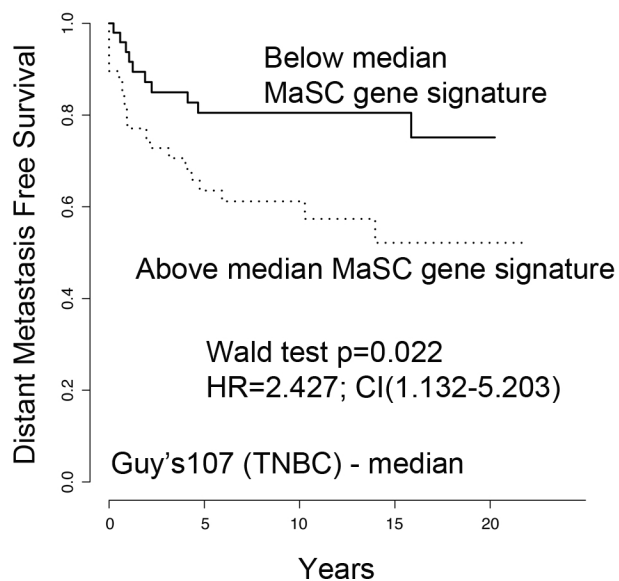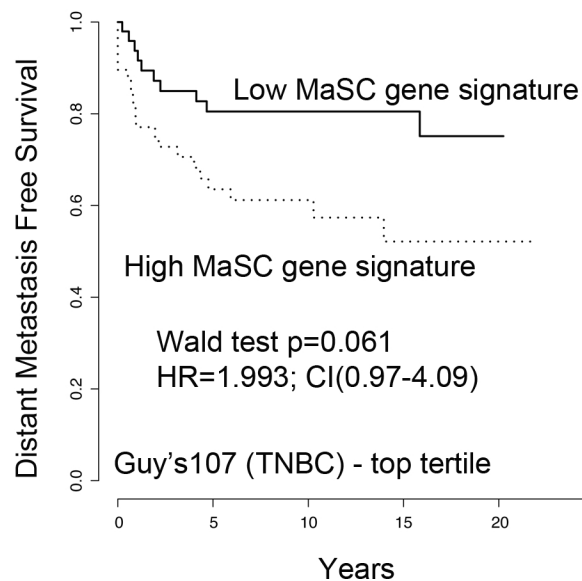

Supplement: Additional file 14: — The MaSC gene signature performs better than random gene lists. (A) Predictive power of 1,000 random gene sets compared to the MaSC gene signature. The frequency of obtained P values is shown in the y-axis, while the -log 10 P values are listed on the x-axis. The black line indicates the P values of the 1,000 gene sets; the P value of the MaSC signature is indicated by the red line. (B) Averaged MaSC signature scores in tumours are correlated with patient outcome, supporting the prognostic power of the DART score. [file 13058_2015_539_MOESM14_ESM.pdf]
