# Supplementary material for: Mouse mammary stem cells express prognostic markers for triple-negative breast cancer
Source: Breast Cancer Res. 2015 Mar 4;17(1):31. doi: 10.1186/s13058-015-0539-6 (PMC4381533; doi:10.1186/s13058-015-0539-6)
Supplement: Additional file 13: — Staining of basal cells for epithelial and mesenchymal markers and heat map of MaSC signature gene expression in Karn579 tumours ordered by DART score. (A) Immunohistochemical staining of two representative regions of mammary epithelium from normal mouse mammary gland for an epithelial (K14) and two mesenchymal (SMA and Vimentin) antigens. Region 1 is an oblique section through the edge of a duct. Bars = 10 um. Note strong staining of basal layer for K14 and SMA, and weaker, punctate staining for Vimentin in a subset of cells (Vimentin-positive cells within the mammary epithelium indicated with an asterisk; the grey dashed line indicates the boundary between the mammary epithelium and the mammary stroma, which includes Vimentin-positive stromal cells). (B) Heat map of expression of the 323 MaSC signature genes in the Karn579 dataset ordered left to right from high DART score tumours to low DART score tumours. Red indicates high expression, green low expression. [file 13058_2015_539_MOESM13_ESM.pdf]

A

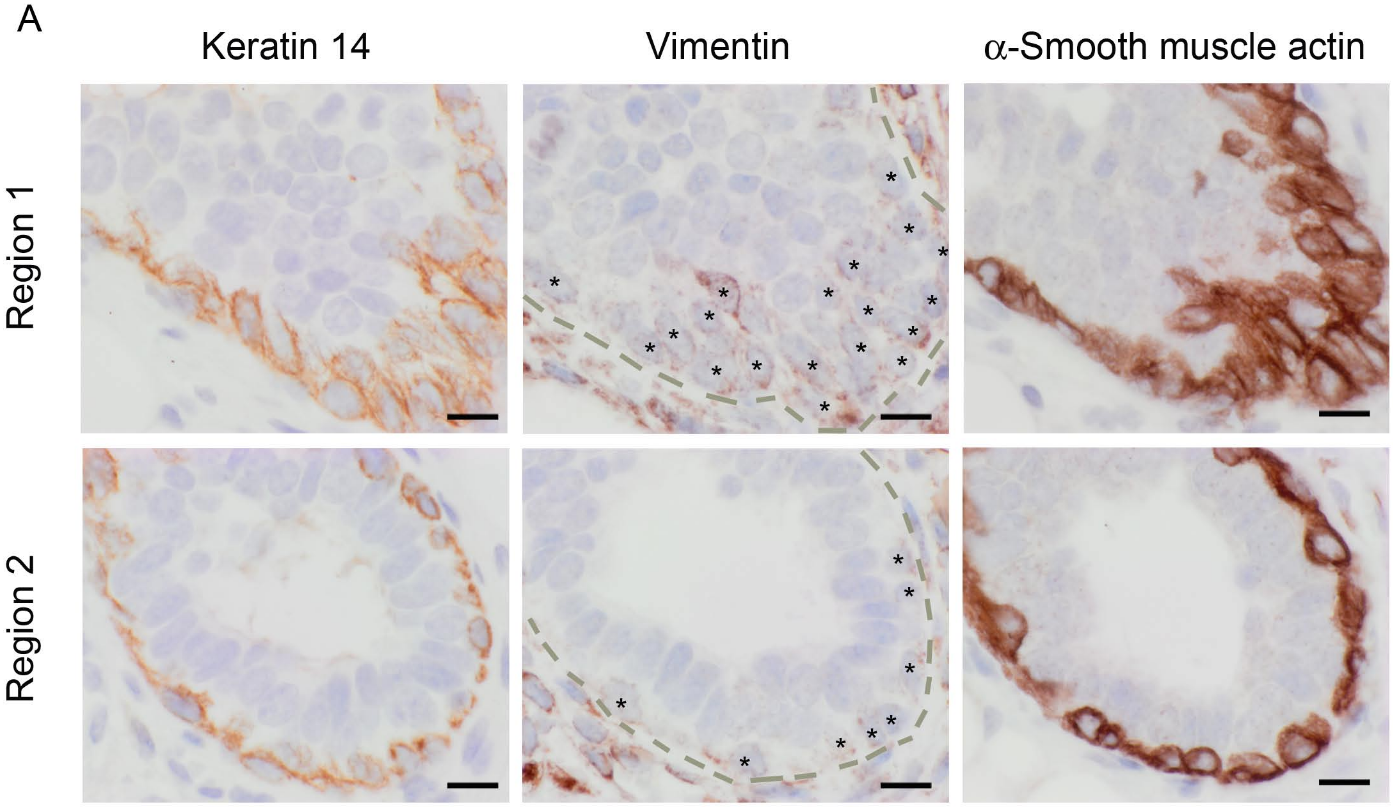
Keratin 14
Vimentin
α-Smooth muscle actin
Region 1
Region 2

B

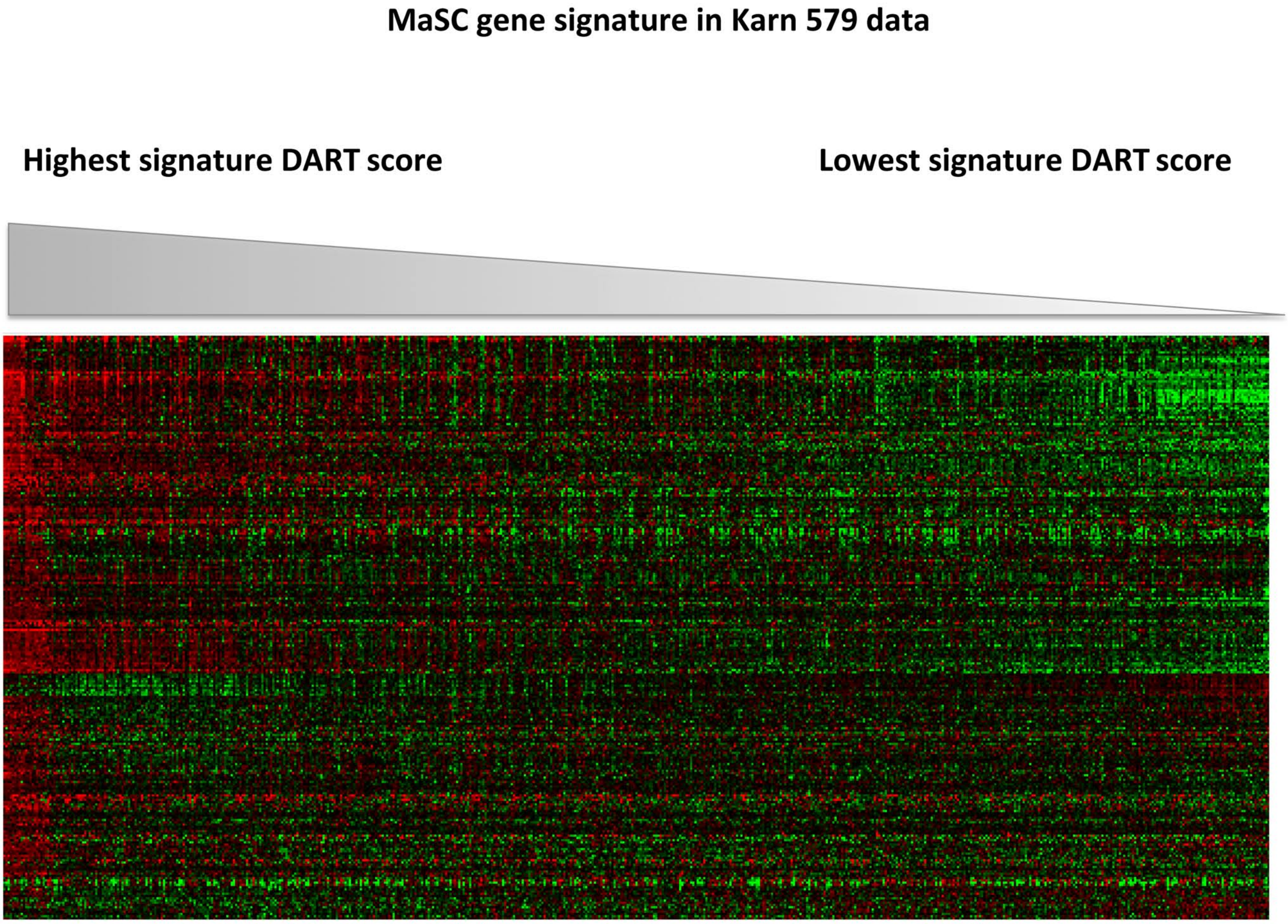
MaSC gene signature in Karn 579 data
Highest signature DART score
Lowest signature DART score
